# Constitutive Contribution by the Rice OsHKT1;4 Na^+^ Transporter to Xylem Sap Desalinization and Low Na^+^ Accumulation in Young Leaves Under Low as High External Na^+^ Conditions

**DOI:** 10.3389/fpls.2020.01130

**Published:** 2020-07-30

**Authors:** Imran Khan, Sonia Mohamed, Thomas Regnault, Delphine Mieulet, Emmanuel Guiderdoni, Hervé Sentenac, Anne-Aliénor Véry

**Affiliations:** ^1^BPMP, Univ Montpellier, CNRS, INRAE, Institut Agro, Montpellier, France; ^2^CIRAD, UMR AGAP, Montpellier, France; ^3^Université de Montpellier, CIRAD, INRAE, Institut Agro, Montpellier, France

**Keywords:** HKT transporters, rice (*Oryza sativa*), OsHKT1;4 Na^+^ transporter, artificial microRNA, xylem sap desalinization, salt tolerance, low Na^+^ growth conditions, Na^+^ transport affinity

## Abstract

HKT Na^+^ transporters correspond to major salt tolerance QTLs in different plant species and are targets of great interest for breeders. In rice, the HKT family is composed of seven or eight functional genes depending on cultivars. Three rice *HKT* genes, *OsHKT1;1*, *OsHKT1;4* and *OsHKT1;5*, are known to contribute to salt tolerance by reducing Na^+^ accumulation in shoots upon salt stress. Here, we further investigate the mechanisms by which OsHKT1;4 contributes to this process and extend this analysis to the role of this transporter in plants in presence of low Na^+^ concentrations. By analyzing transgenic rice plants expressing a *GUS* reporter gene construct, we observed that *OsHKT1;4* is mainly expressed in xylem parenchyma in both roots and leaves. Using mutant lines expressing artificial microRNA that selectively reduced *OsHKT1;4* expression, the involvement of OsHKT1;4 in retrieving Na^+^ from the xylem sap in the roots upon salt stress was evidenced. Since *OsHKT1;4* was found to be also well expressed in the roots in absence of salt stress, we extended the analysis of its role when plants were subjected to non-toxic Na^+^ conditions (0.5 and 5 mM). Our finding that the transporter, expressed in *Xenopus* oocytes, displayed a relatively high affinity for Na^+^, just above 1 mM, provided first support to the hypothesis that OsHKT1;4 could have a physiological role at low Na^+^ concentrations. We observed that progressive desalinization of the xylem sap along its ascent to the leaf blades still occurred in plants grown at submillimolar Na^+^ concentration, and that OsHKT1;4 was involved in reducing xylem sap Na^+^ concentration in roots in these conditions too. Its contribution to tissue desalinization from roots to young mature leaf blades appeared to be rather similar in the whole range of explored external Na^+^ concentrations, from submillimolar to salt stress conditions. Our data therefore indicate that HKT transporters can be involved in controlling Na^+^ translocation from roots to shoots in a much wider range of Na^+^ concentrations than previously thought. This asks questions about the roles of such a transporter-mediated maintaining of tissue Na^+^ content gradients in non-toxic conditions.

## Introduction

Na^+^ is quite abundant in the earth crust (around 2.4%), slightly more than K^+^ (Kronzucker et al., 2013; Haynes et al., 2016; Nieves-Cordones et al., 2016). In the biosphere, however, these two alkali cations display very different distributions. K^+^ is the major inorganic cation in the cytoplasm, where its concentration (in the 0.1 M range) is usually several times higher than that of Na^+^. High concentrations of Na^+^ in the cytoplasm are toxic, resulting in deleterious effects on cell metabolism, in particular on photosynthetic activity.

Na^+^ abundance in the soil is largely heterogeneous, ranging from concentrations higher than those in oceans to traces only (Maathuis, 2013), depending, for instance, on the proximity to the sea (where its average content is *ca*. 1 % w/w), human salinization through agricultural practices and irrigation with poor quality water, local geological variations and rainfalls (the mobility of this cation being quite high in the soil).

By inducing both osmotic and ionic stresses, high soil salinity affects crop production. An electrical conductivity (Ec) of saturated soil paste extract with a threshold of 4 dS/m (corresponding to approximately 40 mM NaCl), was used to define saline soils (Chinnusamy et al., 2005). Most crops suffer from salt stress in these conditions. Rice (*Oryza sativa* L.), which is quite sensitive to salinity, already displays a reduction in yield in most cultivars when grown at Ec higher than 2 to 3 dS/m (Grattan et al., 2002; Negrão et al., 2011). Saline soils represent 6 to 10% of the earth’s lands (FAO, 2008).

Molecular determinants of plant salt stress tolerance have been identified through genetic screens/QTL analysis or reverse genetics approaches (Hasegawa et al., 2000; Chinnusamy et al., 2005; Negrão et al., 2011). These determinants concern in particular genes encoding membrane transport systems involved in controlled distribution and compartmentalization of Na^+^ at the tissue, cell or subcellular levels (Shi et al., 2002; Yokoi et al., 2002; Berthomieu et al., 2003; Hauser and Horie, 2010; Jiang et al., 2010; Shabala et al., 2015; Wu et al., 2015). Among them, Na^+^ transporter genes from the HKT family correspond to salt tolerance QTLs in various species, e.g., different cereals, vine, tomato (Ren et al., 2005; Huang et al., 2006; Byrt et al., 2007; Asins et al., 2013; Campbell et al., 2017; Hazzouri et al., 2018; Henderson et al., 2018; Jiang et al., 2018). In rice, at least three different *HKT* genes, *OsHKT1;5*, *OsHKT1;4* and *OsHKT1;1*, play a role in salt tolerance (Ren et al., 2005; Wang et al., 2015; Suzuki et al., 2016). All three genes contribute to the control of shoot Na^+^ accumulation upon salt stress: *OsHKT1;1*, expressed in phloem and xylem tissues (in both root and leaves; Jabnoune et al., 2009; Wang et al., 2015) allows both a reduced Na^+^ export to the leaves *via* the xylem sap and a higher Na^+^ recirculation to the roots *via* the phloem sap upon salt stress, which favors root *versus* leaf Na^+^ accumulation (Wang et al., 2015; Campbell et al., 2017). *OsHKT1;5*, essentially expressed in root xylem tissues upon salt stress at vegetative stage, controls Na^+^ export to the leaves by desalinizing the xylem sap (Ren et al., 2005; Kobayashi et al., 2017). *OsHKT1;4* has also been shown to reduce Na^+^ accumulation in shoots (especially in reproductive tissue) upon salt stress (Suzuki et al., 2016; Oda et al., 2018). Its expression pattern at the tissue level and the mechanism by which it controls shoot Na^+^ accumulation upon salt stress has however still to be specified.

Outside salt stress conditions, the role of HKT transporters is a lot less known. In conditions of K^+^ deficiency, where low/moderate concentrations of Na^+^ are known to be able to contribute to turgor building and growth in a number of plant species including rice (Yoshida and Castaneda, 1969; Wakeel et al., 2011; Kronzucker et al., 2013), OsHKT2;1, which is involved in root Na^+^ uptake, was shown through the analysis of knock-out mutants to play a role in plant biomass production (Horie et al., 2007). *OsHKT2;1* gene was also identified as likely candidate for a QTL of K^+^ use efficiency in low-K^+^-growth condition, correlating with high shoot Na^+^ content (Miyamoto et al., 2012; Hartley et al., 2020). It should be noted that in quite salt tolerant species like barley, similar *HKT*-mediated beneficial (likely osmotic) effects of Na^+^ on plant growth were noticed, although in K^+^-replete and high saline conditions, upon overexpression of the *HKT2;1* barley gene (Mian et al., 2011).

Overall, however, there is still little information at non-toxic Na^+^ concentrations on the control of Na^+^ transport in the plant, its physiological role, and the possible contribution of HKT transporters to the control of these transports. Here, we were interested in the control of Na^+^ translocation between roots and leaves, with particular interest in non-toxic Na^+^ conditions under standard K^+^ nutrition. We show that the control of Na^+^ delivery to the shoots *via* desalinization of the xylem sap is not restricted to salt tress conditions, and that the rice Na^+^ transporter OsHKT1;4 plays an important role in this constitutive mechanism of xylem sap desalinization.

## Materials and Methods

### Production of amiRNA Lines Displaying Reduced *OsHKT1;4* Transcript Levels

Two artificial microRNA (amiRNA) sequences targeting *OsHKT1;4* transcript (*I3amiR* and *I4amiR*) were selected among those proposed by the WMD3 program (web MicroRNA designer; http://wmd3.weigelworld.org/). Their hybridization energy (indicative of their efficiency) was as recommended (i.e., between −35 and −38 kcal.mol^−1^) for *I4amiR* and slightly higher than that recommended for *I3amiR* (**Table S1**). *I3amiR* and *I4amiR* sequences displayed at most 68% identity with other transcripts than the *OsHKT1;4* target. The hybridization energy of both *I3amiR* and *I4amiR* on these off-target mRNA was thus far below the range given by the software for efficiency of the amiRNA, indicating that substantial off-target effect of the two amiRNA *in planta* was unlikely. Among these weakly likely off-targets was identified a sequence of *OsHKT1;3* mRNA, which showed 66% of identity with the complementary sequence of the *I4amiR*. Each selected 21 mer amiRNA sequence (**Table S1**) was then entered to the program “Oligo” tool which generated four oligonucleotide primers for PCR cloning of the amiRNA precursor DNA sequence to introduce in transgenic plants for producing amiRNA (**Table S2**). The PCR aimed at replacing in a cloned DNA sequence naturally transcribed into a stem-loop precursor of miRNA (*osa-MIR528*), the sequence corresponding to the active 21 nt miRNA and that corresponding to the complementary miRNA* (not strictly complementary to the active 21 nt miRNA sequence on the precursor stem, and degraded during maturation process) by those corresponding to the *I3* or *I4* amiRNA and the corresponding amiRNA* designed by the WMD3 program, targeting *OsHKT1;4*. For both *I3* and *I4*, the construction of amiRNA precursor DNA sequence involved different fragment amplifications, comprising (i) the amiRNA* (replacing *osa-MIR528* miRNA*) and the 3’ region of *osa-MIR528* precursor, (ii) the 5’ region of *osa-MIR528* precursor and the amiRNA (replacing *osa-MIR528* miRNA), and (iii) the amiRNA, the loop region of *osa-MIR528* precursor and the amiRNA* (**Table S2**). A fusion PCR was then performed to synthesize the complete DNA sequences of the *I3* and *I4* stem-loop amiRNA precursors from the three fragments (for each precursor) previously amplified. The fusion PCR product was cloned (Gateway recombination) into pCAMBIA 5300 OVER EXPRESSION vector (Breitler JC, unpublished), placing the DNA sequence of the amiRNA precursor under control of the regulatory region of the maize *ubiquitin 1* gene (**Figure 5A**). Both constructs were sequence verified and introduced in rice cv Nipponbare *via Agrobacterium tumefaciens*. In parallel, some calluses were also transformed with the empty pCAMBIA 5300 OVER EXPRESSION vector for “wild type” transformed plant production.

Rice transformation was performed using the *A. tumefaciens* EHA105 strain, as described by Sallaud et al. (2003). Briefly, dehulled rice seeds were rinsed in ethanol 70% for 90 s and treated with disinfection solution (50 g/l of sodium hypochlorite) for 30 min. After sterilization, the seeds were transferred to a callus induction medium leading to callus development from the scutellum region. Developing calluses were incubated with bacteria suspension for 15 min, blot dried on Whatman paper and co-cultured during 3 days. After the co-culture period, calluses were transferred onto a first medium allowing both selection (hygromycin) of transformed cells and decontamination of calluses (cefotaxime and vancomycin) from agrobacteria, then 15 days later to a second selection medium for 15 days of further growth. Hygromycin-resistant cell lines were transferred to a maturation medium for one week, then allowed to regenerate under light onto a regeneration medium. Four-week-old plantlets (from 19 to 24 independently transformed calluses for each construction) were transferred to soil and grown in greenhouse for seed production.

The reduction in the level of *OsHKT1;4* transcript accumulation in regenerated plants was analysed from the T0 generation in order to discard the less promising lines. For that, RT-PCR experiments on third leaf tissues were carried out to compare the level of *OsHKT1;4* retro-transcripts in amiRNA and wild type plants from the same transformation. Real time PCR primers hybridized upstream of the relative location on *OsHKT1;4* transcript of the amiRNA hybridization (in I3 lines) or on either sides of the relative location on transcript of the amiRNA hybridization (in I4 lines) (**Tables S1** and **S3**).

### Production of Transgenic Plants Expressing the *GUS* Reporter Gene and Histochemical Analysis of GUS Activity

Nipponbare genomic DNA was used to amplify a 2.4-kb fragment of *OsHKT1;4* promoter region by PCR (**Table S4**). A promoter fragment of 2.17 kb was cloned into the pCAMBIA 1301 binary vector upstream the *GUS* reporter gene where it replaced the *CAMV35S* promoter (after addition of *Nco*I cloning sites at the 3’ end by PCR; **Table S4**). Calluses from wild-type rice (cv Nipponbare) grains were transformed *via A. tumefaciens* as described above (selection of transgenic tissues by hygromycin). Plants were regenerated from transformed calluses. Before the transfer of regenerated plant to the greenhouse, samples from leaves and roots were collected to check GUS activity in transformed lines by histochemical analyses. Six plants (obtained from five independently transformed calluses) selected in T0 generation on the basis of highest GUS activity were amplified for further analyses in the T1 generation.

For histochemical analysis of GUS activity, rice tissues from 10 day-old plants (grown in Petri dishes containing 50 mg/l hygromycin), were incubated overnight at 37°C in 50 mM phosphate buffer at pH 7, supplemented with 0.5 mM ferricyanide, 0.05% (v/v) Triton X100 and 1 mM X-Gluc (5-bromo-4-chloro-3-indolyl-ß-D-glucuronide). They were then washed in phosphate buffer, and fixed in GUS-FIX buffer (phosphate buffer containing 1.6% of paraformaldehyde and 0.5% of glutaraldehyde) for 2 h at 4°C under hood. Samples were then dehydrated by successive incubations in water–ethanol solutions containing increasing contents of ethanol (from 50 to 100%), and finally stored at 4°C. The coloration resulting from the GUS activity was observed under binocular magnifier or microscope. For the microscopic observations, tissues were included in a resin (2-hydroxyethyl methacrylate; Technovit 7100, Hereaus-Kulzer GmBH, Wehrheim, Germany), according to the indications of the supplier, and slices of 8 µm thickness were obtained by means of an ultra-microtome (Leica RM2165, Germany) equipped with glass knives. Microscopic section observation was made using a BH2 microscope (Olympus) under white light.

### Plant Growth Conditions for Physiological Analyses

Seeds from wild type rice (*O. sativa* L.) cv Nipponbare or from transgenic rice plants issued from cv Nipponbare were stripped of their husks, disinfected in a 15% bleach solution for 30 min under continuous agitation (100 revolutions/min), rinsed thoroughly, then germinated in Petri dishes on autoclaved Whatman filter paper moistened with sterile water, in a culture chamber (28°C/25°C 14 h/10 h day/night, light intensity of 500 µE m^−2^ s ^−1^). For selection of *Agrobacterium*-mediated transformed plants, seedlings were transferred after three days of germination to new Petri dishes containing 8 ml of hygromycin at 50 mg/l. After six days of antibiotic treatment homo and hemizygous plants for the transgene were either transferred to the greenhouse for seed production or onto hydroponics solutions for physiological experiments. Untransformed wild type plants used in parallel in physiological experiments or for expression studies by qRT-PCR were kept in water for the same time.

After 10 days of germination in Petri dishes, plantlets were cultivated on Yoshida hydroponic solution (0.7 mM KNO_3_, 1.2 mM Ca(NO_3_)_2_, 1.6 mM MgSO_4_, 0.5 mM (NH_4_)_2_SO_4_, 0.8 mM KH_2_PO_4_, 60 µM Na_2_FeEDTA, 20 µM MnSO_4_, 0.32 µM (NH_4_)_6_Mo_7_O_24_, 1.4 µM ZnSO_4_, 1.6 µM CuSO_4_ and 45.2 µM H_3_BO_3_, pH adjusted to 5.5 with H_2_SO_4_) in 8 l containers. The medium was oxygenated by bubbling air and was renewed after 9 days, then every 4 or 5 days. Hydroponic cultures were held in long-day conditions in a growth chamber as for germination (28°C/25°C 14 h/10 h day/night, light intensity of 500 µE m^−2^ s^−1^; relative humidity: 70%). After 12 to 14 days in Yoshida medium, plants were subjected to different salt treatments by transferring them to the Yoshida medium supplemented with the respective NaCl concentrations, or were maintained on Yoshida medium (assayed to contain 0.3 mM Na^+^ by flame photometry). At the end of the treatments, plants displayed (depending on the treatment) three or four expanded leaves.

### Real Time PCR Analyses

Total RNA were extracted from pools of root or shoot tissues of hydroponically grown plants (as described above), using the “RNeasy Mini Kit” (Qiagen, France). Each pool constituted one biological replicate, and at least three biological replicates were done per condition. RNA were treated by DNase using the “DNAse I” kit (Invitrogen), then assayed using Ribogreen reagent (from “Quant-iT™ RiboGreen^®^ RNA” kit; Molecular Probes, France) and a plate reader (VICTOR3 multilabel Counter 1420, Perkin-Elmer, Wellesley, USA). First strand cDNA synthesis was performed from 3 µg of DNase-treated RNA using the “SuperScript III Reverse transcriptase” kit (Invitrogen). Real-time PCR was performed in 96-well plates using the LightCycler^®^ 480 Real-Time PCR System (Roche diagnostics) and SYBR Green I probe (Roche) to monitor cDNA amplification. In each well, the final reaction medium was prepared by mixing 3 µl of cDNA (obtained from 40 ng of DNase-treated RNA), 0.5 µl of each primer (at 10 µM), 5 µl of LightCycler^®^ FastStart DNA Master PLUS SYBR Green I mix (Roche diagnostics) and 1 µl of purified water. All samples were analyzed in triplicates. Applied PCR program (software: LightCycler^®^ 480 1.5; Roche) consisted of 10 min at 95°C, then 45 amplification cycles composed of 10 s at 95°C, 10 s at 60°C, 15 s at 72°C, then a melting curve building (5 s at 95°C, 1 min at 65°C and 5 min with temperature increase from 65 to 97°C), and finally 30 s cooling at 40°C. The absolute number of *HKT* cDNA copies was determined in each sample based on standard curves obtained from dilution series of known amounts of corresponding cDNA fragments. Three housekeeping genes *Tip41* (Caldana et al., 2007), *SMT3* ubiquitin-like protein gene (Horie et al., 2007) and *EF1β* elongation factor gene were used for rough *HKT* expression values normalization, using geNORM v.3.5 software (Vandesompele et al., 2002). Forward and reverse primers used for real-time PCR experiments are given in **Table S3**.

### Na^+^ and K^+^ Assays in Tissues and Xylem Sap

The root system was excised, rinsed in deionized water, then briefly dried in-between two layers of towel-type paper and transferred into pre-weighed plastic vials (20 ml “liquid scintillation” type). Sheaths and blades of third leaves were also collected, separately, and transferred into pre-weighed plastic vials. The samples were dried at 60°C for 3 days and the plastic vials were weighed again, allowing to determine sample dry weight (DW). Na^+^ and K^+^ ions were extracted from dried tissues by solubilizing in 0.1 N hydrochloric acid for 24 h and assayed by flame spectrophotometry (SpectrAA 220 FS, Varian).

The xylem sap was collected by exudation after cutting the rice plants either just above the crown (“hypocotyl region”) or in leaf sheaths at the height of the top of the first leaf sheath, while the roots were still in the nutritive medium. A device consisting of a small container adapted from a pipette tip (Gilson type 1 ml; base diameter slightly superior to that of the base of the root system or sheaths) was sealed around the cut tissue through the application of a polyvinylsiloxane joint (low viscosity elastomer, President Microsystem Light refill body, Coltene). Ten to thirty microliters of sap exuded into this device was transferred (using a micropipette) into a pre-weighed tube (0.2 ml Eppendorf type). Plants having undergone a salt treatment with high NaCl concentrations (addition of 50 or 80 mM NaCl to Yoshida medium) were transferred back onto the Yoshida medium after installation of the sap-collecting devices, the low osmolarity of the Yoshida medium rendering possible sap exudation from excised tissues. Na^+^ concentration in xylem sap samples was determined by flame spectrophotometry.

### Expression in *Xenopus laevis* Oocytes and Two-Electrode Voltage-Clamp

*OsHKT1;4* cDNA, provided by the NIAS (Japan) in pFLCI vector, was amplified by PCR and transferred into the TOPO TA Cloning vector and thereafter into the pGEMXho vector (derived from pGEMDG; D. Becker, Würzburg) downstream from the T7 promoter and between the 5’ and 3’ untranslated regions of the *Xenopus β-globin* gene, by enzymatic restriction using *Not*I and *Hin*dIII sites. Capped and polyadenylated cRNA was synthesized *in vitro* from linearized vector using the mMESSAGE mMACHINE T7 kit (Ambion, Austin, USA), and purified by phenol/chloroform extraction and isopropyl alcohol precipitation. Stage V or VI *X. laevis* oocytes were injected (Nanolitre 2000 injector, World Precision Instruments, USA) with 50 ng of *OsHKT1;4* cRNA in 50 nl of DEPC treated water or with 50 nl of DEPC-treated water (for control oocytes), and then kept at 19°C in “ND96” medium (96 mM NaCl, 2 mM KCl, 1.8 mM CaCl_2_, 1 mM MgCl_2_, 2.5 mM Na-pyruvate, and 5 mM HEPES-NaOH, pH 7.4) supplemented with 0.5 mg.l^-1^ gentamycin. Whole oocyte currents were recorded using the two-electrode voltage clamp technique 1 to 2 days after cRNA injection, as described by Mian et al. (2011). The voltage-clamp amplifier was a GeneClamp 500B (Axon Instruments, Foster City, USA). Voltage-pulse protocols, data acquisition, and data analyses were performed using pClamp10 (Axon Instruments) and Sigmaplot10 (Jandel Scientific, Germany) softwares. Both membrane potential and current were recorded. Correction was made for voltage drop through the series resistance of the bath and the reference electrode using, in addition to the reference electrode, a voltage recording electrode in the bath close to the oocyte surface, both external electrodes being connected to a bath probe (V6-2A ×100 virtual ground bath clamp; Axon Instruments). All electrodes were filled with 3 M KCl. The external solution bathing the oocyte was continuously percolated during the voltage-clamp experiment.

The bath solutions contained a background of 6 mM MgCl_2_, 1.8 mM CaCl_2_, and 10 mM MES-1,3-bis[tris(hydroxymethyl)methylamino]propane (BTP), pH 5.5. Monovalent cations were added to the background solution as glutamate salts. The chloride concentration was constant in each set of solutions. D-Mannitol was added to adjust the osmolarity (same osmolarity in each set of solutions in the range 220–240 mosM). For the analysis, the current passing through OsHKT1;4 was determined by subtracting from the total current recorded in the oocyte expressing OsHKT1;4, the average of endogenous currents in the same solution of percolation recorded in three to five control oocytes (injected with water).

## Results

### A Desalinization Mechanism of the Xylem Sap From Root to Blade in Rice Similarly Operates in High and Low Na^+^ Conditions

**Figure 1:**
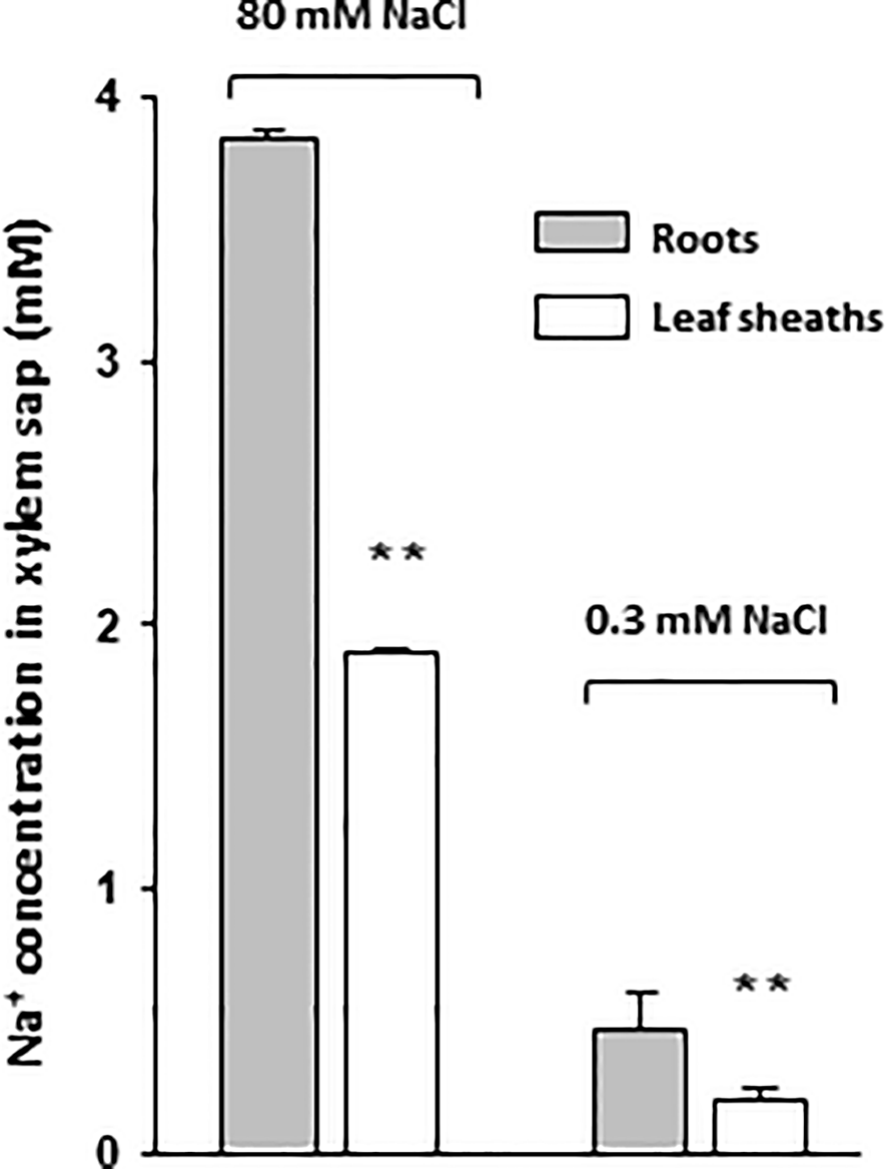
Sodium concentration in xylem sap exudates of roots and leaf sheaths of rice plants treated with high or low NaCl concentrations. Xylem sap exudates were collected following excision of aerial plant organs either at the leaf base (root exudate; grey bars) or at the level of the top of the sheath of the first leaf (sheath exudate; white bars). Na^+^ content of the hydroponic growth medium was either 0.3 mM during 14 days or 0.3 mM during 14 days then 80 mM during 2 days (plant growth and treatment conditions of **Figure S1A**). Excised plants treated with 80 mM NaCl were transferred back to 0.3 mM Na^+^-containing medium to allow exudation. Data: means ± SE (n = 3 to 6). Two stars above bars of leaf sheath exudates indicate significant difference with root exudates according to Student t-test (P ≤ 0.01).

Na^+^ concentration in the xylem sap arriving to the leaves is a strong determinant of Na^+^ accumulation in leaf tissues (*e.g.*, in rice: Yeo et al., 1985). In cereals treated with high NaCl concentrations, a lower Na^+^ concentration in xylem sap has been linked to increased salt tolerance (Ren et al., 2005; James et al., 2006), and desalinization processes of xylem sap along its ascent to the tip of the leaves have been reported to play crucial roles in maintaining low Na^+^ concentration in young blade tissues (Wolf et al., 1991). In rice, in which a large apoplastic bypass flow exists in the root leading to rather poor Na^+^ exclusion (Yeo et al., 1987; Flam-Shepherd et al., 2018), efficient mechanisms of Na^+^ extraction from the xylem sap is likely of particular importance. In **Figure 1**, the change in Na^+^ concentration in xylem sap along its ascent to the blades of the leaves was compared in rice cv Nipponbare plants in their fourth week of growth, either treated with high Na^+^ concentration (plants grown on Yoshida hydroponic medium supplemented with 80 mM NaCl during two days) or grown at low Na^+^ concentration (Yoshida medium containing 0.3 mM Na^+^) (**Figure S1A**). In plants grown for two days at 80 mM NaCl, a decrease (by about a factor of 2) in xylem sap Na^+^ concentration was found between the extremity of the root system and the top of the first leaf sheath. Interestingly, a very similar decrease (also by about a factor of 2) was observed in plants grown in the presence of 0.3 mM Na^+^, although the Na^+^ concentration in xylem sap was very low in these conditions (9 times lower than in 80 mM Na^+^-treated plant exudates; ~0.45 mM for the sap collected just above the root system). These results indicated that the desalinization of the xylem sap along its ascending path from roots to shoots is a constitutive mechanism, which takes place, apparently to a similar extent, in high and low Na^+^ conditions, in wild type rice plants.

### *HKT* Na^+^ Transporter Expression in Rice Tissues in Standard Conditions and Upon High Salt Treatment

**Figure 2:**
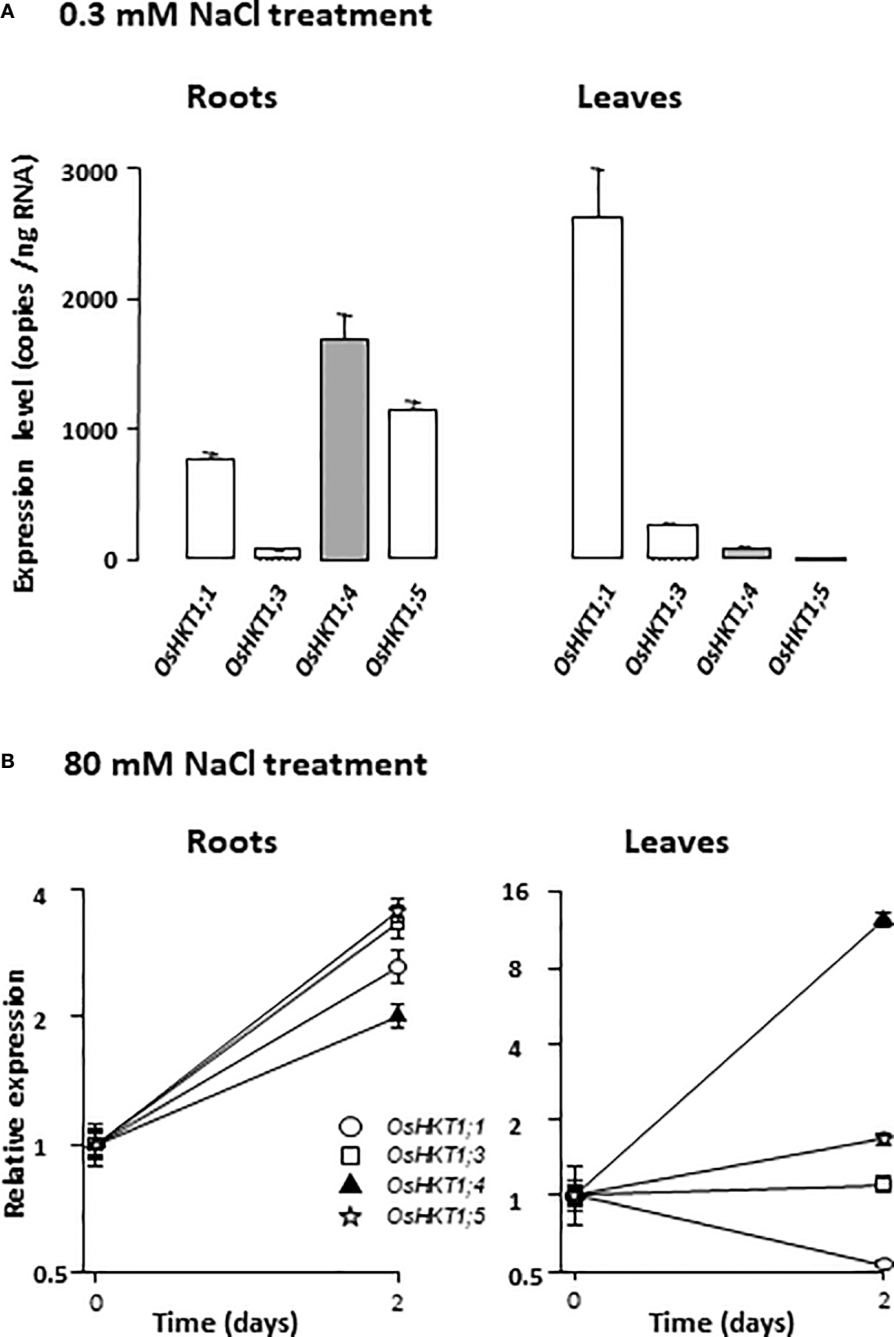
Expression levels of HKT Na^+^ transporter genes in rice plants in low or high NaCl conditions. **(A)** Expression levels in plants hydroponically grown on Yoshida medium (containing 0.3 mM Na^+^). Real-time RT-PCR were performed on 40 ng of total RNA isolated either from root pools (left) or leaf pools (right) of 22 day-old plants (four plants in each pool). Expression data of the different HKT members of subfamily 1, given in absolute copy number per ng of RNA, are mean values (± SE) of three biological replicates. *Tip41* and *SMT3* constitutive genes were used as controls for calibration of qRT-PCR experiments (*Cf. Materials and Methods*). **(B)** Salt stress effect on *HKT* gene expression in rice roots (left) and leaves (right). *HKT* gene expression was compared in plants grown as in **(A)** or subsequently subjected to salt stress by exposure for 2 days to 80 mM NaCl added to the hydroponic Yoshida medium. Real-time RT-PCR was performed as in **(A)**. Relative expression: for each gene, the level of expression is reported to the mean expression level determined in plants grown in absence of salt stress (data from panel **A**). Means ± SE of three biological replicates (y axis: binary logarithmic scale).

Several Na^+^ transporters from the HKT family have been shown to play important roles in decreasing xylem sap Na^+^ concentration in salt stress conditions (Hauser and Horie, 2010). With the aim to investigate the possible role of HKT Na^+^ transporters in rice xylem sap desalinization in low Na^+^ conditions, the levels of expression of the rice *HKT* genes from subfamily 1, which encode Na^+^-selective transporters (Platten et al., 2006), were analyzed in rice cv Nipponbare plants grown in the presence of low Na^+^ concentration (0.3 mM Na^+^ in Yoshida medium) and compared with those in salt stressed plants (same batch of plants subsequently subjected to a 80 mM NaCl treatment for two days). The Nipponbare *HKT* subfamily 1 comprises four members, *OsHKT1;1*, *OsHKT1;3*, *OsHKT1;4* and *OsHKT1;5* (the *OsHKT1;2* sequence corresponding to a pseudogene; Garciadeblás et al., 2003). The plants were in their fourth week of growth, as in the experiment in **Figure 1**, when sampled and analyzed. In plants grown in presence of 0.3 mM Na^+^, real-time RT-PCR analyses indicated that, amongst the four members of the HKT subfamily 1 in the Nipponbare cultivar, *OsHKT1;4* was the one that displayed the most abundant transcript in roots (reaching 1700 copies per ng of RNAs; **Figure 2A** left). *OsHKT1;5* and *OsHKT1;1* were a bit less expressed (30 and 55% less, respectively), and the expression of *OsHKT1;3* was very low. In the leaves of these plants grown in low Na^+^ conditions, *OsHKT1;1* was highly expressed as compared to the other sub-family 1 *HKT* genes (**Figure 2A** right). Following *OsHKT1;1*, the most expressed *HKT* member in leaves in these standard culture conditions was *OsHKT1;3*, displaying about 9% of *OsHKT1;1* expression level. The other members had particularly low expression levels in leaves in standard conditions (**Figure 2A** right).

When the plants were treated for 2 days with 80 mM NaCl in their growth medium, the expression of all subfamily 1 *HKT* members was increased in roots (**Figure 2B** left). The increase in expression was however modest (by a factor of 2 to 3). The lowest increase was observed for *OsHKT1;4*. In leaves with the same duration of salt stress application, only *OsHKT1;4* showed a large increase in expression (by a factor >10), the other members of subfamily 1 displaying either weak change in expression (for *OsHKT1;3* and *OsHKT1;5*), or a ~2 time decrease (for *OsHKT1;1*), as compared to untreated plants (**Figure 2B** right).

Thus, differences in expression level between *HKT* genes were observed in both roots and leaves. The fact that some *HKT* members were observed to be well expressed not only in salt stress conditions but also in standard growth conditions suggested that these members could have a significant physiological role even in absence of salt stress, in particular in xylem sap desalinization. *OsHKT1;4*, which displayed the highest level of expression in roots in standard conditions, amongst the *HKT* subfamily 1 genes, and whose induction in roots upon salt stress was the weakest, was favored for further studies.

**Figure 3:**
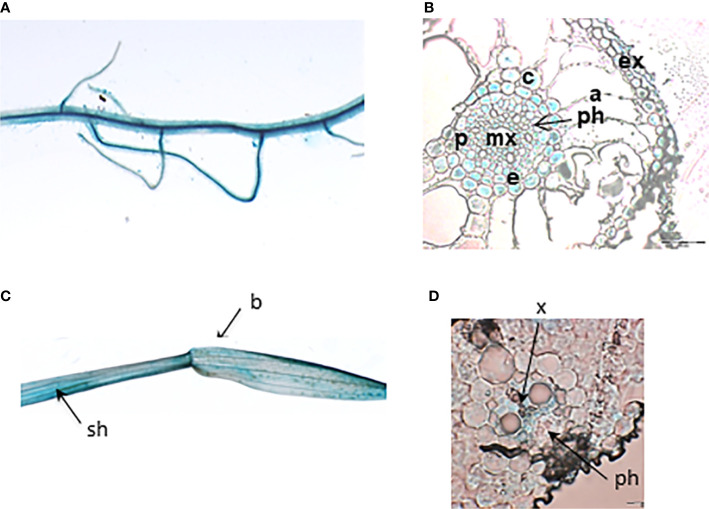
Histochemical analysis of GUS activity controlled by the *OsHKT1;4* promoter region in transgenic rice plants. **(A, B)** Root tissues. **(A)** Main root and lateral roots under binoculars. **(B)** Cross section of root under microscope (bar: 50 µm). **(C)** Leaf sheath and blade under binoculars. **(D)** Blade cross section under microscope (bars: 20 µm). Plants were grown in Petri dishes for 10 days in MS/2 medium **(A, C)** or in distilled water **(B, D)**. a, aerenchyma; b, leaf blade; c, cortex; e, endodermis; ex, exodermis; mx, metaxylem; p, pericycle; ph, phloem; sh, sheath; x, xylem.

In order to determine the expression pattern of *OsHKT1;4*, rice transgenic plants expressing *OsHKT1;4* promoter fused to the *β-glucuronidase* (*GUS*) reporter gene were generated. *OsHKT1;4* promoter activity was analysed in low Na^+^ conditions (**Figure 3**). Strong GUS activity was detected by histochemical tests in the different analysed plants in the main root and secondary roots (**Figure 3A**), and in the leaf, in both the sheath and the blade (**Figure 3C**). Cross sections of root and leaf tissues stained by GUS activity showed that *OsHKT1;4* promoter was active in vascular tissues (mostly xylem parenchyma) of both roots and leaves (**Figures 3B, D**). In roots, it was also well active in peripheral layers (exodermis, cortex, endodermis and pericycle) (**Figure 3B**).

### OsHKT1;4 Na^+^ Transporter Affinity for Na^+^ and Regulation by External K^+^

Some functional properties of OsHKT1;4 have been previously determined following expression of the transporter in yeast and *Xenopus* oocytes (Suzuki et al., 2016). These studies indicated that OsHKT1;4 displays bi-directional transport activity and transports preferentially Na^+^ among monovalent cations. We performed further functional analyses in order to determine the apparent affinity of Na^+^ transport by OsHKT1;4, and to examine possible interactions between K^+^ and Na^+^ upon transport. OsHKT1;4 was expressed in *Xenopus* oocytes and functionally studied using two-electrode voltage clamp.

**Figure 4:**
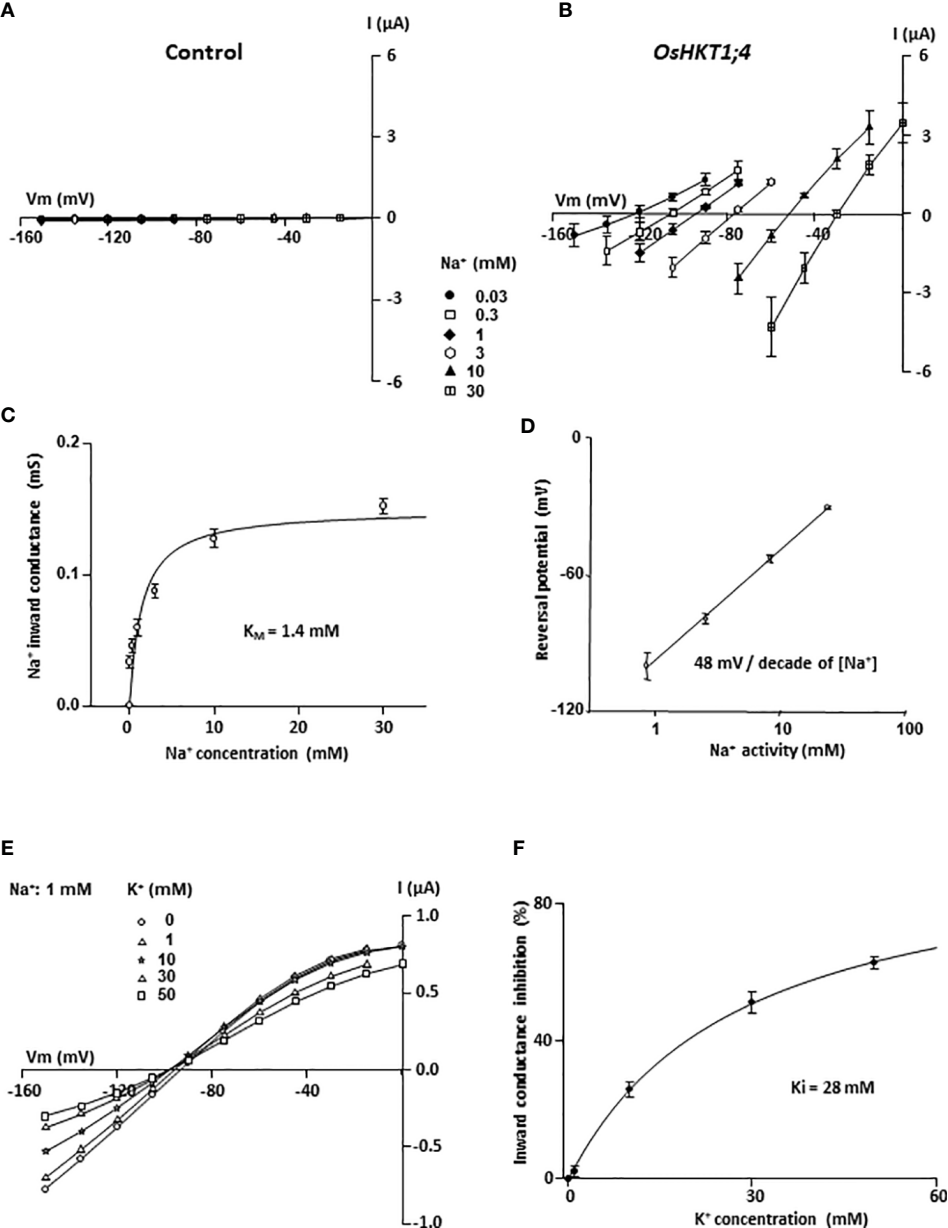
Affinity for Na^+^ and sensitivity to K^+^ of OsHKT1;4 transporter expressed in *Xenopus* oocytes. **(A, B)** Effect of external Na^+^ concentration (glutamate salt) in the range of 0.03 to 30 mM on current–voltage (I–V) relationships in control (water injected) oocytes **(A)** and in OsHKT1;4-expressing oocytes **(B)**. The current passing through OsHKT1;4 was determined by subtracting the mean current in water-injected oocytes from the whole oocyte current in each OsHKT1;4-expressing oocyte. Data are means ± SE (n = 4) and are representative of two experiments performed in different oocyte batches. **(C)** OsHKT1;4 apparent affinity for Na^+^. Na^+^ inward conductance: slope of the I–V relationship derived from the three imposed potentials that were the closest to the reversal potential. The Na^+^ concentration at which half saturation of the inward conductance occurred (apparent K_M_), determined with a hyperbolic fit (Michaelis–Menten equation) on mean conductance data was 1.4 mM. Data are means ± SE (n = 4). **(D)** Sensitivity of OsHKT1;4 reversal potential of current to external Na^+^ activity. The logarithmic fit of OsHKT1;4 reversal potential of current change with external sodium activity had a slope of 48 mV/decade of sodium activity at external Na^+^ ≥1 mM. Data are means ± SE (n = 4). **(E, F)** Inhibition of OsHKT1;4 sodium transport by potassium. **(E)** Current–voltage relationships of OsHKT1;4-mediated currents in a representative oocyte in bath solutions containing 1 mM Na-glutamate and varying concentrations of K-glutamate (0, 1, 10, 30 or 50 mM). The current passing through OsHKT1;4 was determined by subtracting the mean current in water-injected oocytes from the whole oocyte current in each OsHKT1;4-expressing oocyte. **(F)** OsHKT1;4 inward conductance inhibition by K^+^. OsHKT1;4 conductance was determined close to the reversal potential (between –120 and –150 mV). The relative inhibition of OsHKT1;4 conductance by K^+^ was calculated for each tested concentration of external K^+^, as the difference between the conductance in the absence of K^+^ (G in 0 K^+^) and the conductance in the presence of the tested concentration of K^+^, expressed as percent of G in 0 K^+^. A hyperbolic Michaelis–Menten equation adjusted to the data (means ± SE, n = 5) gave an apparent inhibition constant, Ki, of 28 mM.

To determine the affinity of OsHKT1;4 for Na^+^, different concentrations of Na^+^ in the external medium were used, that varied from 0.03 to 30 mM (**Figure 4B**). Positive shifts in the reversal potential of OsHKT1;4 currents and increases in OsHKT1;4 conductance were observed as the concentration of Na^+^ in the bath solution was increased, as expected for a non-rectifying sodium transporter (**Figure 4B**; Suzuki et al., 2016). With the aim to perform quantitative analyses, the current recorded in OsHKT1;4-expressing oocytes was compared with that in control (water-injected) oocytes in the same conditions (same oocyte batch, ionic condition and voltage applied), to check that the current through OsHKT1;4 was dominant (at least 10 times higher than the endogenous oocyte current), even at low Na^+^ concentrations (**Figures 4A, B**, **Figures S2A, B**). Then, the current passing through OsHKT1;4 was extracted from the whole OsHKT1;4-expressing oocyte current by subtracting the mean current recorded in control oocytes in the same conditions (**Figure 4B**). Plotting OsHKT1;4 inward conductance *versus* the Na^+^ external concentration showed that OsHKT1;4 conductance increased in a saturable manner with the Na^+^ concentration (**Figure 4C**). The concentration at which half saturation of the inward conductance occurred (apparent K_M_) was determined with a hyperbola fit to be 1.4 mM (**Figure 4C**). This indicated that OsHKT1;4 is a low-affinity Na^+^ transporter, but suggested that its activity in xylem tissues may be already significant in plants grown in standard conditions displaying several hundred µM of Na^+^ in xylem sap (**Figure 1**).

The reversal potentials of OsHKT1;4 currents in the solutions varying in Na^+^ concentration in the 1 to 30 mM range changed by 48 mV when the external Na^+^ activity increased by 10-fold (**Figure 4D**). This was close to a Nernstian behavior (58 mV per 10-fold change in the external Na^+^ activity expected for a purely Na^+^-selective channel), as also noticed by Suzuki et al. (2016), and confirmed that Na^+^ was the main ion passing through OsHKT1;4 in these conditions. The selectivity against K^+^ of OsHKT1;4 was estimated by comparing OsHKT1;4 inward conductance in bath solutions containing successively NaCl and KCl at 10 mM, and by analyzing the shift in the reversal potential of OsHKT1;4 currents upon cation change (**Figure S2**). Using the Goldman–Hodgkin–Katz formalism, a permeability ratio P_Na_/P_K_ of 4 was obtained (**Figures S2C, D**). The mean inward conductance ratio G_Na_/G_K_ was determined to be of 2.5 (**Figure S2E**). Other monovalent cations (Rb^+^, Cs^+^, Li^+^) did not display significant difference in permeability and conductance when compared with K^+^ (**Figure S2**). These quantifications suggested that, although OsHKT1;4 displays clear selectivity for Na^+^, K^+^ transport through this system may not be negligible in certain ionic situations, like strong external concentration differences in favor to K^+^.

The effect of external K^+^ concentration increase on OsHKT1;4 currents in the presence of a low (fixed) concentration of Na^+^ was investigated (**Figure 4E**). K^+^ concentration was successively 0, 1, 10, 30 and 50 mM, and Na^+^ concentration was 1 mM. The currents mediated by OsHKT1;4 were reduced when the external K^+^ concentration was increased (**Figure 4E**). The inward current was more affected. However, the outward current appeared also reduced when K^+^ concentration reached 30 mM. On the other hand, no positive shift in the reversal potential of OsHKT1;4 currents upon K^+^ increase was observed, indicating that when Na^+^ and K^+^ were both present in the external medium, K^+^ behaved rather as a blocker than as a permeant ion in OsHKT1;4. The percentage of inhibition by external K^+^ of OsHKT1;4 inward conductance was plotted *versus* the external K^+^ concentration and was fitted to a Michaelis–Menten equation to determine the concentration of K^+^ producing half inhibition (Ki). The mean Ki was found to be 28 mM (**Figure 4F**).

### Production of Transgenic Rice Lines Displaying Reduced Expression of *OsHKT1;*4 Using Artificial MicroRNAs

**Figure 5:**
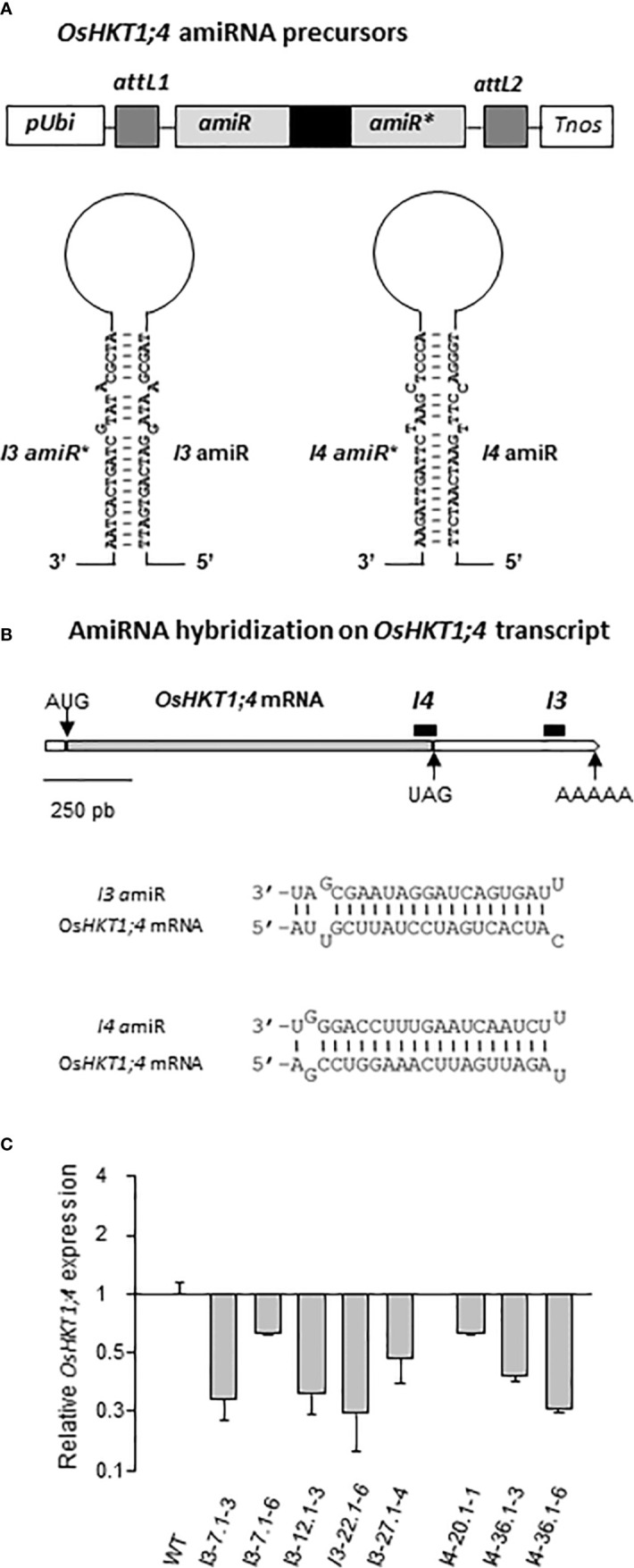
Production of transgenic rice lines overexpressing artificial microRNA precursors in order to knock-down the expression of *OsHKT1;4*. (**A** Top) Schematic representation of the two constructs cloned in the pCambia5300 vector to allow overexpression of artificial microRNA degrading *OsHKT1;4* transcripts in transgenic rice plants. *pUbi*: promoter of *Arabidopsis thaliana ubiquitin* gene; *attL1*, *attL2*: recombinant sequences allowing the cloning (using the Gateway system) of sequences whose transcription produces the precursor of artificial microRNA. *Tnos*: *NOS* transcription terminator. (**A** Bottom) Representation of the 2 artificial microRNA precursors *PI3* (left panel) and *PI4* (right panel) transformed in rice. Maturation of these precursors leads to the active microRNA *I3amiR* and *I4amiR*, respectively. The transformed lines were named I3-# and I4-#, respectively. (**B** Top) Position of hybridization of the microRNA *I3amiR* and *I4amiR* (black bars) on *OsHKT1;4* mRNA. (**B** Bottom) Hybridization with two mismatches of *I3amiR* and *I4amiR* on *OsHKT1;4* mRNA. **(C)** Relative expression of *OsHKT1;4* in *oshkt1;4* amiRNA rice lines I3-# and I4-# from the T2 generation. Control plants (WT) are issued from the same transformation as I3-# and I4-# lines, but do not express *PI3* or *PI4* microRNA precursors. The expression of *elongation factor 1-beta* (*EF-1-beta*) was used as a constitutive calibration control in qRT-PCR experiments. Results from five plants issued from a same T1 plant were averaged (Means ± SE).

Conception of artificial microRNA (amiRNA) based on natural miRNA biogenesis and action is one of the biotechnology tools that have been used to specifically decrease expression of a gene of interest (Schwab et al., 2006; Warthmann et al., 2008). Such strategy was used to produce mutant lines with reduced expression of *OsHKT1;4*, since no mutant with an insertion in the coding sequence of this gene was available in the international collections. A single and stable amiRNA (as the miRNA) is expected to be processed from a single-stranded precursor transcript displaying a stem-loop structure with imperfect foldbacks (Kurihara and Watanabe, 2004; **Figure 5A**). The active single stranded 21-bp amiRNA (amiR), derived from the stem of the precursor guides the cleavage and degradation of the 16target RNA, through hybridization. Production of amiRNA has been reported as a highly effective and sequence specific approach for post transcriptional gene silencing in plants (Schwab et al., 2006). The imperfect complementary microRNA to the amiR released from the stem of the precursor (amiR*) is degraded during the maturation process.

A dedicated web platform (web MicroRNA designer; http://wmd3.weigelworld.org/) was used to design amiR sequences targeting *OsHKT1;4* transcript (and the “complementary” amiR* sequences), and to assess the quality of the proposed amiR (efficiency, specificity). Two proposed amiRNA, that were named *I3amiR* and *I4amiR*, were chosen (**Figures 5A, B**; **Table S1**). Their hybridization position on *OsHKT1;4* mRNA was at the very end of the translated region (*I4amiR*) and within the 3’UTR region (*I3amiR*) (**Figure 5B** top; **Table S1**). Both amiRNA displayed two hybridization mismatches on *OsHKT1;4* transcript at the position of their 1st (*I3amiR* and *I4amiR*) and 19th (*I3amiR*) or 20th (*I4amiR*) nucleotides (**Table S1**, **Figure 5B** bottom).

Rice plants (cv Nipponbare) were transformed to overexpress either of the cloned amiRNA precursors including the I3amiR-I3amiR* or I4amiR-I4amiR* duplex sequences (**Figure 5A**). Some of the calluses were also transformed with the “empty” vector to obtain transformed plants unaffected in *OsHKT1;4* expression as control.

Some “*I3*” and “*I4*” plants were selected among those regenerated, based on expression of *OsHKT1;4*. Progenies of eight out of 11 T1 plants (issued from five and two different calluses transformed with *I3* and *I4* amiRNA precursors, respectively) displayed a reduction in *OsHKT1;4* expression by at least 40% and up to 70%, when compared with the expression in control plants (**Figure 5C**). Analysis of the expression of the other *HKT* Na^+^ transporter genes in the I3 and I4 plants indicated that the *I3* and *I4* amiRNA specifically affected the expression of *OsHKT1;4* (**Figure S3**). The expression of neither *OsHKT1;1*, *OsHKT1;3* nor *OsHKT1;5* was significantly reduced in these *I3* and *I4* amiRNA lines. The expression of these other *HKT* Na^+^ transporter genes was not induced either, except in one *I4* line for *OsHKT1;1* (showing ~3-fold induction) and two *I3* lines for *OsHKT1;5* (showing ~2-fold induction).

### Role of OsHKT1;4 in Rice Young Leaf Desalinization in a Large Range of Na^+^ Concentration Conditions

In salt stress conditions, the ability to maintain low Na^+^ content in young leaf blade tissues is recognized as a major adaptive mechanism in salt sensitive cereals like rice (Greenway and Munns, 1980; Platten et al., 2013; Munns et al., 2016). We investigated the young leaf blade desalinization in rice plants treated with varying Na^+^ concentrations, high (80 mM for 2 days), moderate (5 mM for 5 days) and low (0.3 mM for 2 weeks). The high and moderate Na^+^ concentration treatments were performed on 3-week-old and 4-week-old plants, respectively (**Figure S1A**). The plants in the low Na^+^ concentration treatment were 3-week old at sampling (**Figure S1A**). Both *oshkt1;4* amiRNA mutant and wild type plants were included in this experiment, the latter ones being essentially untransformed plants (cv Nipponbare). A few transformed plants with the empty vector were also included, which allowed to check that the transformation process had not induced significant changes in tissue Na^+^ accumulation behavior (**Figure S4**).

**Figure 6:**
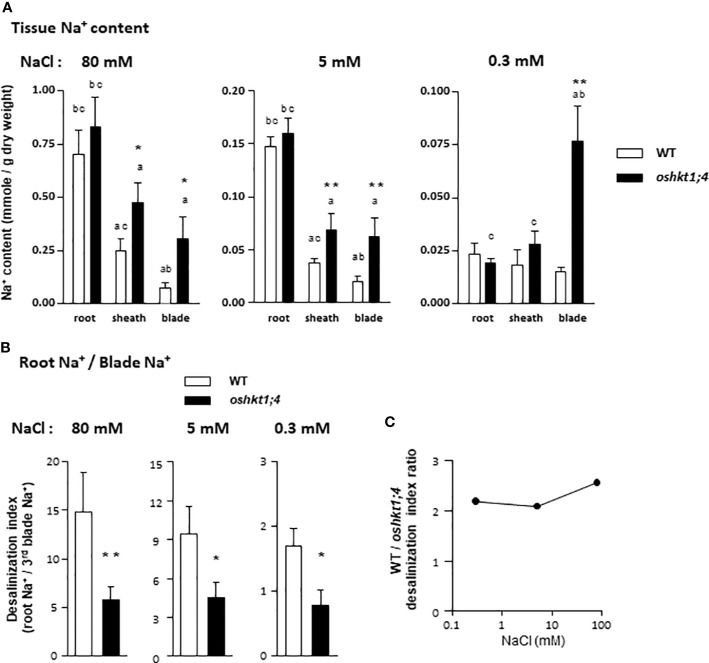
Sodium contents in roots and in leaf tissues of rice wild type and *oshkt1;4* knockdown mutant plants treated with different levels of salt. Plants were grown and treated as described in **Figure S1A**. Mutant plants displaying reduced expression of *OsHKT1;4* (**Figure 5C**) were issued from T2 generation of amiRNA I3-# and I4-# lines. **(A)** Sodium contents in the roots, the sheath of the third leaf and the blade of the third leaf of plants treated with 80 mM NaCl (left panel), 5 mM NaCl (middle panel) or 0.3 mM NaCl (right panel). Means ± SE. Plants treated with 80 mM NaCl: n = 8 for the WT plants and 21 for the *oshkt1;4* mutant plants. Treatment with 5 mM NaCl: n = 7 and 13 for the WT and the *oshkt1;4* plants. Treatment with 0.3 mM NaCl: n = 11 and 21 for the WT and the *oshkt1;4* plants. One or two stars above a bar of mutant plants indicates that the difference with the corresponding wild type (WT) plants is statistically significant according to a Student t-test (P ≤0.05 or ≤0.01, respectively). A letter above a bar (a, b or c) indicates a significant difference with another tissue (respectively root, leaf sheath or leaf blade) within a same genotype (Student t-test, P ≤0.05). **(B)** Roots to third leaf blade desalinization index: ratio of the root Na^+^ content to the blade Na^+^ content of the same plant. Plants were from the same experiment as in **(A)**, treated with either 80 mM NaCl (left panel), 5 mM NaCl (middle panel) or 0.3 mM NaCl (right panel). Means ± SE. Treatment with 80 mM NaCl: n = 11 for the WT plants and 27 for the *oshkt1;4* mutant plants. Treatment with 5 mM NaCl: n = 7 and 13 for the WT and the *oshkt1;4* plants. Treatment with 0.3 mM NaCl: n = 14 and 24 for the WT and the *oshkt1;4* plants. One or two stars above a bar of mutant plants has the same meaning as in **(A)**. **(C)** Comparison of the efficiency of desalinization (from roots to third blade) between WT and *osht1;4* mutant plants grown under the three salt treatments. The ratios of the mean value of the roots to third blade desalinization index displayed by the wild type plants to that displayed by the *osht1;4* mutant plants [data from **(B)**] are plotted against the treatment salt concentration, 0.3, 5 or 80 mM NaCl.

In wild type plants, the root Na^+^ contents were lower by respectively about 5-folds and 30-folds, in plants treated with 5 mM and 0.3 mM Na^+^ than in plants treated with 80 mM Na^+^ (**Figure 6A**). The Na^+^ contents in 3rd leaf sheaths and blades of wild type plants also decreased with the decrease in Na^+^ concentration in the treatment medium, although to a lesser extent than in root tissues, e.g., by about 5-folds between the 80 and 0.3 mM Na^+^ treatments (**Figure 6A**).

In wild type plants treated with 80 mM or 5 mM NaCl, the Na^+^ content was significantly lower in the sheath of the 3rd leaf than in the roots, and in the blade of the 3rd leaf than in the sheath of the same leaf (**Figure 6A**), indicating occurrence of tissue desalinization process. A desalinization index was defined as the ratio of the root Na^+^ content to the third leaf blade Na^+^ content of the same plant. This desalinization index was found to be not very different in plants treated with 80 or 5 mM Na^+^, being respectively close to 15 and 10 (**Figure 6B**), although Na^+^ tissue contents were 4 to 6-fold different between the two types of plants. In the 0.3 mM Na^+^ treatment, the desalinization index was lower, close to 1.7 (**Figure 6B**).

The role of OsHKT1;4 in young leaf desalinization was examined through the comparison of tissue Na^+^ contents in the *oshkt1;4* amiRNA plants included along with wild type plant in the experiment. The *oshkt1;4* amiRNA plants belonged to the progeny of the eight T1 plants selected for their selective reduced level of *OsHKT1;4* expression (**Figure 5C** and **Figure S3**). Having checked that *oshkt1;4* amiRNA plants expressing *I3* or *I4* constructs displayed similar tissue Na^+^ accumulation phenotypes (**Figure S4**), data obtained with the different amiRNA lines were subsequently indistinctly pooled as *oshkt1;4* mutant data. Analysing altogether different mutant lines also allowed to “buffer” the slight induction of other *HKT* Na^+^ transporter gene expression that occurred in a few plants (**Figure S3**).

In *oshkt1;4* mutant plants treated with high or moderate Na^+^ concentrations, desalinization of the young leaf tissues still occurred (**Figure 6A**). Indeed, Na^+^ accumulation in the 3rd leaf in these conditions was still significantly lower than that in the roots. However, the desalinization of aerial tissues in *oshkt1;4* mutant plants in these conditions (80 or 5 mM Na^+^ treatments) was not as effective as that in the wild type plants. Indeed, while Na^+^ contents in roots were not significantly different between wild type and *oshkt1;4* plants, Na^+^ contents in the sheath and the blade of the 3rd leaf were significantly higher in the mutants, by about 2 folds in the sheath and more than 3 folds in the blade (**Figure 6A**). It should be noted that the fact that globally higher Na^+^ contents were detected in the analyzed tissues of *oshkt1;4* plants suggested a possible additional defect in Na^+^ compartmentalization between young and old leaves or higher root Na^+^ uptake in the mutant. The desalinization index (ratio of root Na^+^ content to third leaf blade Na^+^ content; *Cf*. above) was strongly lower in *oshkt1;4* mutant than in wild type plants, by a factor close to 2 to 2.5 in plants treated with 80 or 5 mM Na^+^ (**Figures 6B, C**). When submitted to the low Na^+^ treatment (growth in presence of 0.3 mM NaCl), *oshkt1;4* mutant plants did not display roots to young blade tissue desalinization any longer. In contrast, Na^+^ contents were higher in the third leaf blade than in the roots (**Figure 6A**). In this treatment, the desalinization index was ca. two times lower in the mutant than in the wild type plants, as for the other Na^+^ treatments (**Figures 6B, C**). Thus, OsHKT1;4 was strongly involved in reducing Na^+^ accumulation in young blade tissues even when growth occurred in presence of external Na^+^ concentrations as low as 0.3 mM.

### Role of OsHKT1;4 in Control of the K^+^ to Na^+^ Balance in Young Leaves

**Figure 7:**
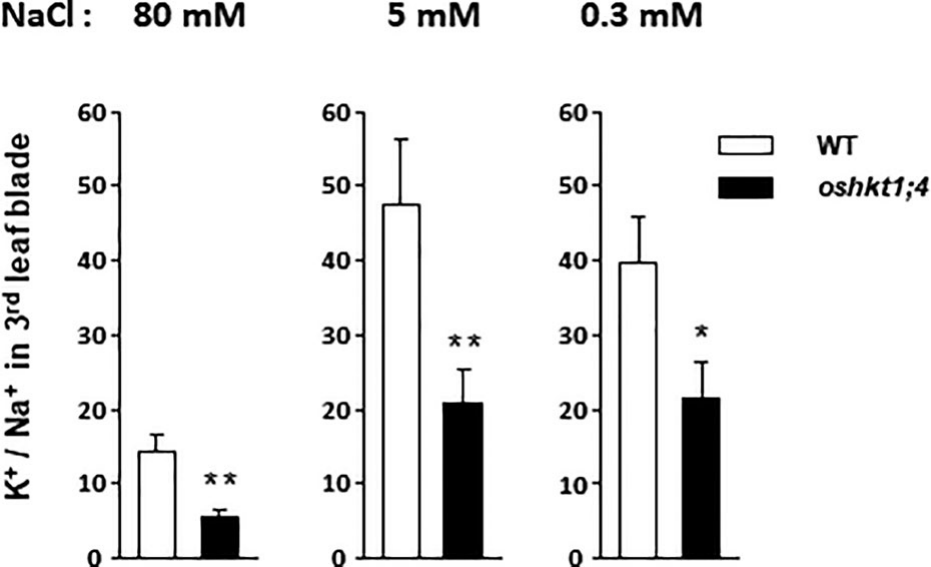
K^+^/Na^+^ ratios in the third leaf blade of rice wild type and of *ohkt1;4* knock-down mutant plants treated with high or low sodium concentrations. Plants are from the same experiment as those analyzed in **Figure 6**, treated with either 80 mM NaCl (left panel), 5 mM NaCl (middle panel), or 0.3 mM NaCl (right panel). Each blade sample was assayed for the contents in both K^+^ and Na^+^ and the corresponding K^+^/Na^+^ ratio was determined. Plants treated with 80 mM NaCl: n = 10 for the WT plants and 27 for the *oshkt1;4* mutant plants. Treatment with 5 mM NaCl: n = 7 and 13 for the WT and the *oshkt1;4* plants. Treatment with 0.3 mM NaCl: n = 13 and 25 for the WT and the *oshkt1;4* plants. One or two stars above a bar of *oshkt1;4* mutant plants indicate that the difference with the corresponding wild type (WT) plants is statistically significant according to a Student t-test (P ≤0.05 or ≤0.01, respectively).

In contrast to the Na^+^ contents, the K^+^ contents of the plant tissues appeared quite independent of the applied Na^+^ treatment in the wild type as well as in the *oshkt1;4* mutant plants (**Figure S5**). Salt stress is generally reported to induce decrease in tissue K^+^ contents in salt sensitive species like rice (Hussain et al., 2017; Liu et al., 2019). Here however, a short duration of the salt stress treatment, applied on well-developed 4-week old plants likely explains the absence of significant effect on tissue K^+^ contents. The ratio of K^+^ to Na^+^ contents in leaves is classically considered as a physiological indicator of salt stress intensity and also of the level of plant tolerance to salinity (Chhipa and Lal, 1995; Maathuis and Amtmann, 1999). The plant ability to maintain this ratio to a high value in young leaves when growth occurs in presence of high external Na^+^ concentrations is indeed positively correlated with the ability to tolerate high salinity (Hauser and Horie, 2010). The K^+^ contents of the roots and of the sheath and blade of the third leaves were, in contrast to the Na^+^ contents, not significantly different in *oshkt1;4* mutant and in wild type plants, whatever the treatment, in high (80 mM), moderate (5 mM) or low (0.3 mM) Na^+^ external concentrations (**Figure S5**). The ratio of K^+^ to Na^+^ contents in the third leaf blade was determined in wild-type and *oshkt1;4* mutant plants subjected to the three treatments in order to evaluate the role of OsHKT1;4 in rice young leaf K^+^/Na^+^ homeostasis. In wild type plants, this ratio was high and similar in plants treated with moderate or low Na^+^ concentrations, close to 40 and 47, respectively, and much lower, by 3–3.5 times, in plants treated with the high Na^+^ concentration (**Figure 7**). In each of these three conditions, the ratio was strongly lower in *oshkt1;4* mutant plants than in wild type plants, by about 2-folds (2.5, 2.2 and 1.9-folds when the plants were treated with high, moderate and low Na^+^ concentrations, respectively; **Figure 7**).

### Involvement of OsHKT1;4 in Xylem Sap Desalinization in High and Low Na^+^ Conditions

**Figure 8:**
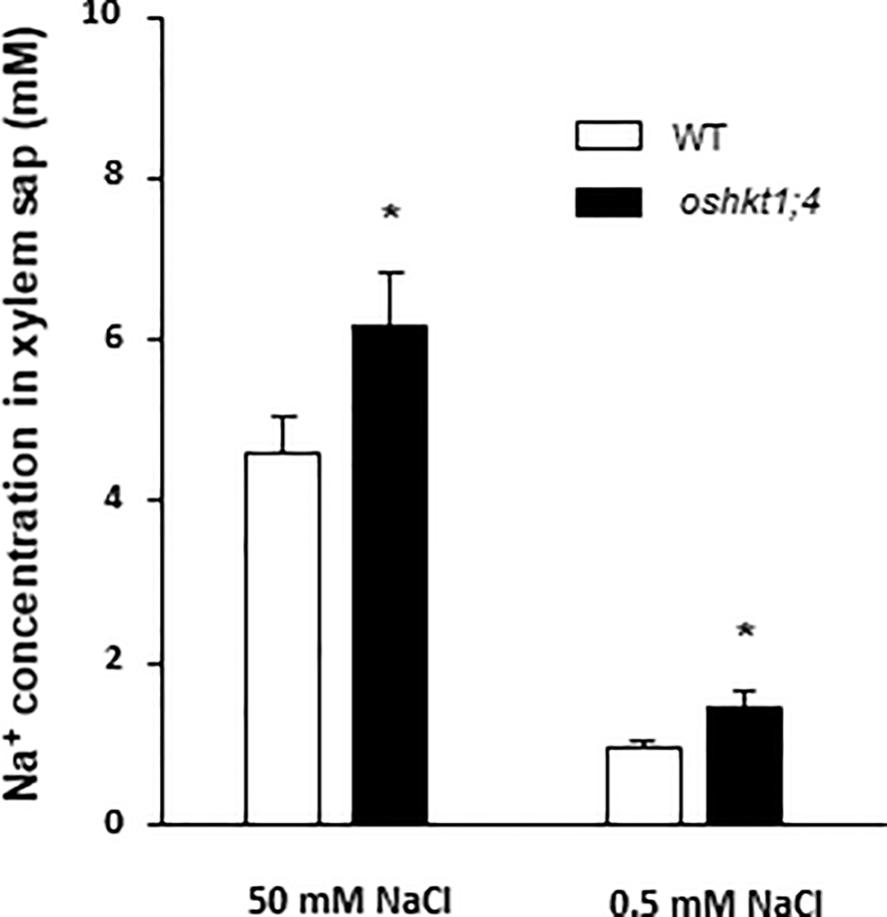
Effect of knockdown expression of *OsHKT1;4* on the Na^+^ concentration in xylem sap exudates of rice plants treated with high or low NaCl concentrations. Wild type plants and *oshkt1;4* amiRNA plants of the T2 generation were grown and treated as in **Figure S1B**: Na^+^ concentration of the hydroponics medium was 0.3 mM during 14 days, then either 0.5 mM during 6 days or 0.5 mM during 3 days then 50 mM during 3 days (solution background: Yoshida medium). Xylem sap was collected by exudation following excision of aerial plant tissues at the leaf base. Excised plants treated with 50 mM NaCl were transferred back to the 0.5 mM Na^+^-containing medium to allow exudation. Xylem sap Na^+^ concentration data obtained in three independent experiments were pooled and are presented as means ± SE (n = 15 to 38). The stars indicate that the corresponding differences between the WT and *oshkt1;4* mutant plants are statistically significant according to Student t-test (P ≤0.05).

Na^+^ accumulation in shoots is thought to be essentially controlled by the delivery of Na^+^ *via* the xylem sap (Munns and Tester, 2008). The role of OsHKT1;4 in controlling xylem sap Na^+^ concentration was investigated at high and low external Na^+^ concentrations. Three-week-old wild type and *oshkt1;4* mutant plants hydroponically grown were used (**Figure S1B**). The high Na^+^ treated plants received 50 mM NaCl for 3 days. The NaCl concentration was fixed at a lower value (but was applied for a longer time) than that (80 mM) in the previous experiment where Na^+^ tissue contents were analyzed, in order to facilitate xylem sap exudate collection. The low Na^+^ treated plants were grown in the presence of 0.5 mM Na^+^ for 6 days. The xylem sap collected after shoot excision (at the base of the leaves) had more than four times higher Na^+^ concentration in high Na^+^ treated plants than in low Na^+^ treated ones (**Figure 8**). In both conditions, Na^+^ concentration in collected xylem sap was significantly higher in *oshkt1;4* mutant plants than in wild type plants, by 35 and 60% in the plants treated with the high and low Na^+^ concentration, respectively (**Figure 8**). This indicated that OsHKT1;4 plays a substantial role in xylem sap desalinization in a large range of external Na^+^ conditions.

## Discussion

### Characterization of OsHKT1;4: Differences and Similarities With Other Subfamility 1 Na^+^-Selective Rice Transporters

The HKT family includes more members and is more diverse, with the presence of two subfamilies, in cereals than in dicots (Platten et al., 2006; Véry et al., 2014). In rice cv Nipponbare, it includes seven predicted functional members (Garciadeblás et al., 2003). Here, we were interested in subfamily 1, which has been shown to contribute to Na^+^ exclusion from young leaves upon salinity stress (Hamamoto et al., 2015), and which comprises four functional genes in cv Nipponbare, *OsHKT1;1*, *OsHKT1;3*, *OsHKT1;4* and *OsHKT1;5*. Among them, *OsHKT1;4* had to be further characterized, with respect to its tissue expression pattern and functional properties of the transporter it encodes.

Our analyzes indicate that the expression of *OsHKT1;4* is mainly localized in the vascular tissues (**Figure 3**), like that of the other members of the subfamily 1 (Ren et al., 2005; Jabnoune et al., 2009; Wang et al., 2015). The expression pattern of *OsHKT1;4* in the vascular tissues of the roots and leaves, which largely includes xylem tissues, is very similar to that of *OsHKT1;5* (Ren et al., 2005; Kobayashi et al., 2017). On the other hand, that of *OsHKT1;1* and *OsHKT1;3* comprises more phloem tissues (Jabnoune et al., 2009; Wang et al., 2015). *OsHKT1;4*, in absence of salt stress, is more expressed in the roots than in the leaves, like *OsHKT1;5* and in contrast to the two other rice subfamily 1 members (**Figure 2A**, Ren et al., 2005; Wang et al., 2015). However, upon salt stress, while the expression of *OsHKT1;5* remains essentially root-specific (Ren et al., 2005), that of *OsHKT1;4* becomes balanced between roots and leaves, due to a strong induction in the leaves (**Figure 2B**).

At the functional level, all the members of subfamily 1 are known to preferentially transport Na^+^ (Corratgé-Faillie et al., 2010). The selectivity among cations is however not very high. The ratio of permeability P_Na_/P_K_ in OsHKT1;4 was determined to be about 4 (**Figure S2**), a bit lower than that determined for OsHKT1;3 (Sassi et al., 2012). Important differences in terms of affinity for Na^+^ have been pointed out among subfamily 1 HKT members. In rice, the affinity of OsHKT1;3 for Na^+^ is more than 20-folds higher than that of OsHKT1;1 (Jabnoune et al., 2009). We determined that the affinity for Na^+^ of OsHKT1;4 is still 2.5-folds higher than that of OsHKT1;3 (**Figure 4C**; Jabnoune et al., 2009). OsHKT1;4 affinity for Na^+^ is also slightly higher (by 1.5 folds) than that of OsHKT1;5 (Somasundaram et al., 2020). Thus, within the rice HKT subfamily 1, OsHKT1;4 displays the highest affinity for Na^+^. OsHKT1;4 affinity for Na^+^ is also higher than that of the wheat homologs of this system that have been characterized so far (Ben Amar et al., 2014; Tounsi et al., 2016). Differences in affinity and maximal conductance between HKT1;4-type transporters from einkorn and durum wheat have been proposed to contribute to the differences in salt tolerance between these species (Tounsi et al., 2016).

Na^+^ transport by OsHKT1;4 is inhibited in the presence of high concentrations of external K^+^ (**Figure 4E**), like in OsHKT1;1 but unlike in OsHKT1;3 and OsHKT1;5 (Ren et al., 2005; Jabnoune et al., 2009). Owing to the determined value of the inhibition constant (28 mM K^+^ in the presence of 1 mM Na^+^, **Figure 4F**), this regulation is likely to have an impact on the transport activity of OsHKT1;4 *in planta*, in plants growing in presence of low external Na^+^ concentrations and displaying Na^+^ concentrations in the xylem sap in the submillimolar range (**Figure 1**).

Thus, overall, OsHKT1;4 more particularly differs from the other members of subfamily 1 by a higher affinity for Na^+^ and by a strong response of the relative levels of transcript accumulation between roots and aerial organs to Na^+^ concentrations in the external medium. The fact that OsHKT1;4 showed a higher affinity for Na^+^ than other HKT transporters of subfamily 1 and that it was well expressed in the roots under standard culture conditions led us to examine its potential role in controlling the transport of Na^+^ between roots and leaves in a large range of Na^+^ concentration conditions, including non-toxic ones.

### OsHKT1;4 Plays a Constitutive Role in Rice Young Mature Leaf Desalinization Through the Control of Xylem Sap Na^+^ Concentration

Like OsHKT1;5 and OsHKT1;1 (Ren et al., 2005; Wang et al., 2015; Campbell et al., 2017; Kobayashi et al., 2017), OsHKT1;4 plays a role in saline stress tolerance by preventing excessive accumulation of Na^+^ in sensitive aerial parts. It has been shown to “desalinize” varying aerial organs/tissues upon salt stress: young leaves, flag leaf, peduncle, nodes, internodes, stem, and seeds (Suzuki et al., 2016; Oda et al., 2018). The mechanism of leaf and reproductive organ desalinization upon salt stress remains however elusive. The strongest expression levels of *OsHKT1;4* upon one-month salt stress were observed in reproductive tissues (peduncle), internodes and flag leaf sheath (Suzuki et al., 2016). Cotsaftis et al. (2012) proposed a mechanism of Na^+^ exclusion from leaf blades involving retrieval of Na^+^ from the xylem sap by OsHKT1;4 in leaf sheaths and loading with Na^+^ of leaf sheath parenchyma tissues, based on observation of strong expression of *OsHKT1;4* in leaf sheaths upon salt stress. In the inflorescence, indications of a role for OsHKT1;4 in Na^+^ unloading from xylem sap have been obtained *via* ^22^Na^+^-imaging experiments (Suzuki et al. (2016): inflorescence peduncles excised from wild type or *oshkt1;4* knockdown mutant plants and soaked in a ^22^Na^+^ solution displayed a steeper longitudinal gradient of ^22^Na^+^ in the case of the wild type plants. No direct assessment of OsHKT1;4 function in xylem tissues was however available in previous studies since Na^+^ concentration of the xylem sap was not analyzed in *oshkt1;4* mutant plants in any tissue.

Previous analyses on the role of OsHKT1;4 in the plant were performed by analyzing two types of plants affected in *OsHKT1;4* expression: RNAi mutant plants that displayed reduced expression of this gene (Suzuki et al., 2016) or plants that overexpressed *OsHKT1;4* due to a T-DNA tagging-based gain-of-function mutation (Oda et al., 2018). We used another type of mutant plants displaying a reduction in the level of *OsHKT1;4* transcripts, through production of artificial microRNA precursors to be processed by the cell machinery for releasing very specific microRNA targeting *OsHKT1;4* transcripts. We show that the strategy we used specifically targeted *OsHKT1;4* among subfamily 1 *HKTs* (**Figure S3**) and resulted in a reduction in the accumulation level of the targeted transcript similar to that reported for conventional RNAi constructs (**Figure 5C**; Suzuki et al., 2016; Campbell et al., 2017).

Previous analyses of *oshkt1;4* knockdown plants had focused on the flowering stage (Suzuki et al., 2016). At this stage, *OsHKT1;4* expression in wild type plants is higher than at vegetative stage, while expression in the roots upon salt stress is low. We performed our analyses on younger plants where expression of *OsHKT1;4* upon salt stress was quite balanced between roots and leaves (**Figure 2**). In the 3-week-old plants we analyzed, the results revealed a significant involvement of OsHKT1;4 in reducing the accumulation of Na^+^ in blades of young mature leaves under salt stress conditions (**Figure 6**). The hypothesis that this reduction in Na^+^ accumulation involved OsHKT1;4 activity in xylem tissues and desalinization of xylem sap was supported by both the analysis of *OsHKT1;4* expression pattern using a *GUS* reporter gene and xylem sap assays (**Figures 3** and **8**). Upon salt stress, in agreement with the latter hypothesis, the xylem sap collected from roots after shoot excision at the base of the leaves contained more Na^+^ in *oshkt1;4* knockdown mutant plants than in wild type plants (**Figure 8**), which revealed a role for OsHKT1;4 in xylem sap desalinization in the roots in young salt stressed plants.

In wild type plants, we observed that desalinization of the xylem sap along its ascent from roots to leaf blades was not restricted to salt stress conditions but occurred as well in presence of external Na^+^ concentration as low as 0.3 mM (**Figure 1**). In *oshkt1;4* mutant plants, xylem sap Na^+^ concentration at the base of the leaves was higher than in wild type plants, when plants were treated in such low Na^+^ conditions (**Figure 8**). This suggested that in high as in low Na^+^ conditions, OsHKT1;4 controls xylem sap Na^+^ concentration in the roots by a same mechanism, reducing Na^+^ accumulation in young leaves (**Figure 6**). This mechanism probably consists in Na^+^ retrieval from the xylem sap and Na^+^ loading in xylem parenchyma cells. Such a mechanism has been proposed for OsHKT1;5 upon salt stress (Ren et al., 2005; Kobayashi et al., 2017), although direct evidence of Na^+^ loading to xylem parenchyma by, e.g., *in situ* ion concentration imaging is lacking. It should be noted however, that based on expression data (**Figure 2**), while OsHKT1;4-mediated xylem sap desalinization would be mainly restricted to the roots at low Na^+^ concentrations, it would be extended to the leaves (likely essentially leaf sheaths; Suzuki et al., 2016) in salt stress conditions.

In our experiments, the xylem sap concentration at the exit of the roots ranged from 0.45 mM (in plants grown in the presence of 0.3 mM Na^+^; **Figure 1**) to about 4 mM (in short term salt stressed plants; **Figures 1** and **8**). Since xylem sap desalinization occurred in root tissues, the sap Na^+^ concentration in contact with the transporter upstream in the root system can be expected to be higher. We measured that in *oshkt1;4* knock-down plants in which desalinization by OsHKT1;4 was strongly reduced, the xylem sap Na^+^ concentration was 35 to 60% higher than in wild type plants (**Figure 8**). The sap Na^+^ concentration in the roots in contact with the transporter could therefore be hypothesized to be in the 0.5–1 mM range in plants grown at low Na^+^, and roughly 10 times more in salt stressed plants. In plants transferred from low Na^+^ to salt stress conditions, the root to young blade desalinization index strongly increased in wild type plants (**Figure 6C**). Such a response to the onset of salt stress conditions could involve increased OsHKT1;4 Na^+^ transport activity, in response to the increased concentration of Na^+^ in the xylem sap, since this concentration could remain in a range where the transporter did not much saturate (**Figure 4C**). In addition, when external Na^+^ was increased to salt stress conditions, higher Na^+^ transport activity by OsHKT1;4 was also achieved by increasing the level of expression of the transporter and by extending the tissue area of transporter action (**Figure 2**), allowing to further increase the level of desalinization.

OsHKT1;4, by controlling Na^+^ content in young leaf tissues also contributed to control the K^+^/Na^+^ ratio in these tissues (the ratio in the young blades in the different Na^+^ treatments being lower in the mutant plants by at least 50%; **Figure 7**). K^+^/Na^+^ balance in leaf cell cytosol plays an important role in salt stress tolerance and the control of the ratio of K^+^ to Na^+^ exported to the leaves *via* the xylem sap has been evidenced as an important factor of salt stress tolerance (Chen et al., 2007; Shabala et al., 2010). The regulation of OsHKT1;4 activity by external K^+^ (**Figure 4F**) is expected to contribute to adjust the K^+^ to Na^+^ concentration ratio in xylem sap. Therefore, OsHKT1;4, which is quite constitutively expressed with respect to Na^+^ growth conditions may have also a role of “K^+^ sensor” in the xylem sap.

As a whole, beside refining the knowledge on the mechanism by which OsHKT1;4 desalinizes young leaf blades in salt stress conditions, our study evidenced that OsHKT1;4 activity contributes to the desalinization of young leaf blades also when plants are grown in presence of a few millimolar, and even at submillimolar, external Na^+^. This shows that the existence of root-to-blade gradients of Na^+^ tissue contents observed in conditions of moderate as well as low Na^+^ conditions cannot be simply ascribed to rate-limited diffusive mechanisms from roots to shoots or growth-induced dilution in the blades, but involves a transporter-mediated “active” desalinization of the xylem sap, which is shown to strongly contributes to build up such root-to-blade gradients. The physiological meaning of this constitutive desalinization of young mature blade tissues is yet unknown. Is Na^+^ toxicity in rice leaves higher than believed and are cellular K^+^/Na^+^ gradients in the range of 40 to 50 (**Figure 7**) required for optimal blade cell functions? This is not very likely owing to the vacuolar Na^+^ compartmentalization capacities. Nevertheless, the fact that complex combination of transporter properties and regulations (expression level and location control, affinity, activity adjustment by K^+^) exists to build up and control root-to-blade Na^+^ gradients also in conditions of low Na^+^ suggests a role of these gradients *in planta* in a large range of Na^+^ concentrations.

## Data Availability Statement

The raw data supporting the conclusions of this article will be made available by the authors, without undue reservation.

## Author Contributions

A-AV, HS, and IK conceived the original research plans. A-AV supervised the experiments. DM and EG managed transgenic plant production. IK, SM, and TR performed most of the experiments. A-AV, HS, IK, SM, and TR analyzed the data. A-AV and IK wrote the first draft of the manuscript. All authors contributed to the article and approved the submitted version.

## Funding

This work was supported in part by a scholarship from the Higher Education Commission of Pakistan (to IK), by the European Research Area Network Plant Genomics Programme (grant no. ERA–PG FP/06. 018B to HS), and by the Agropolis Fondation under the Rice Functional Genomics platform (Montpellier, France).

## Conflict of Interest

The authors declare that the research was conducted in the absence of any commercial or financial relationships that could be construed as a potential conflict of interest.
