# Supplementary material for: Constitutive Contribution by the Rice OsHKT1;4 Na+ Transporter to Xylem Sap Desalinization and Low Na+ Accumulation in Young Leaves Under Low as High External Na+ Conditions
Source: Front Plant Sci. 2020 Jul 30;11:1130. doi: 10.3389/fpls.2020.01130 (PMC7406799; doi:10.3389/fpls.2020.01130)
Supplement: Supplementary file 1 [file DataSheet_1.pdf]

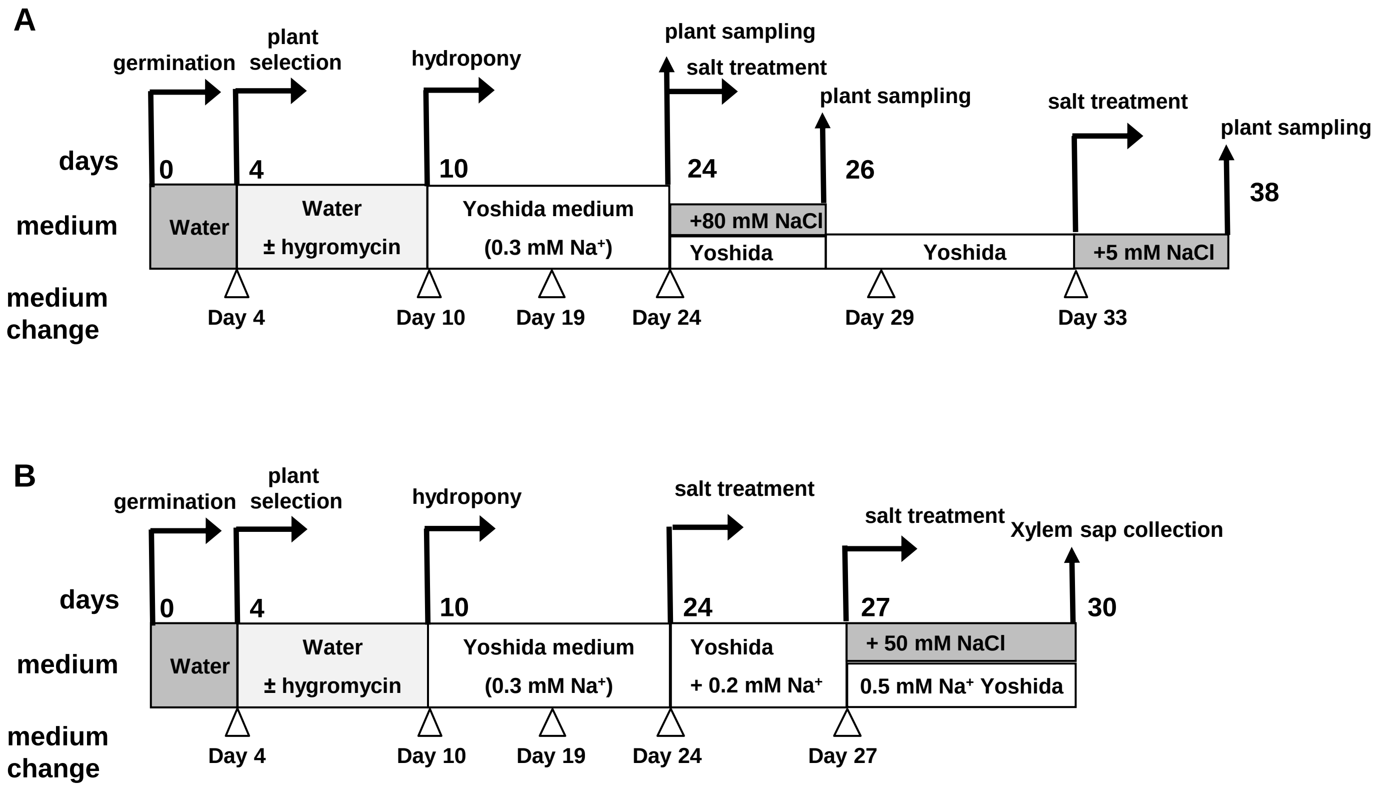

FIGURE S1 | Diagrammatic representation of the physiological experiments performed on *oshkt1;4* amiRNA lines and wild type plants. The scheme shows plant growth conditions (boxes), the times of start of the different growth conditions (right-angled arrows above the boxes), the times of plant material sampling (vertical arrows above the boxes), and the times of medium changes or renewal (triangles below the boxes). Disinfected seeds of both *oshkt1;4* amiRNA and wild-type (transformed with empty vector and untransformed) genotypes were germinatedr in Petri dishes in distilled water. The amiRNA seedlings were then transferred into hygromycin (50 mg/l) to eliminate the wild type plants present in the amiRNA lines (T2 generation). After 10 days, the plants were hydroponically grown on Yoshida medium (containing 0.3 mM Na^+). In the experimental protocol described in (A), after 2 weeks in hydroponics, part of the plants were harvested and the remaining plants were divided into two batches, being transferred onto either Yoshida medium supplemented with 80 mM NaCl (salt treatment) or Yoshida medium. The former plants were submitted to the salt treatment for 2 days and then harvested. The latter plants remained on Yoshida medium for 9 days (until day 33) and were then transferred onto Yoshida medium supplemented with 5 mM NaCl, on which they grew for 5 days before being sampled. In the protocol described in (B), the plants were hydroponically grown for 2 weeks (after germination and, for amiRNA plants, antibiotics selection) like in (A). They were then transferred on Yoshida medium supplemented with 0.2 mM NaCl (final concentration of Na^+: 0.5 mM). After 3 days on this medium, the plants were transferred onto either Yoshida medium supplemented with 50 mM NaCl or 0.5 mM Na^+ Yoshida medium. All the plants were used three days later.
