# Supplementary material for: Constitutive Contribution by the Rice OsHKT1;4 Na+ Transporter to Xylem Sap Desalinization and Low Na+ Accumulation in Young Leaves Under Low as High External Na+ Conditions
Source: Front Plant Sci. 2020 Jul 30;11:1130. doi: 10.3389/fpls.2020.01130 (PMC7406799; doi:10.3389/fpls.2020.01130)
Supplement: Supplementary file 2 [file DataSheet_2.pdf]

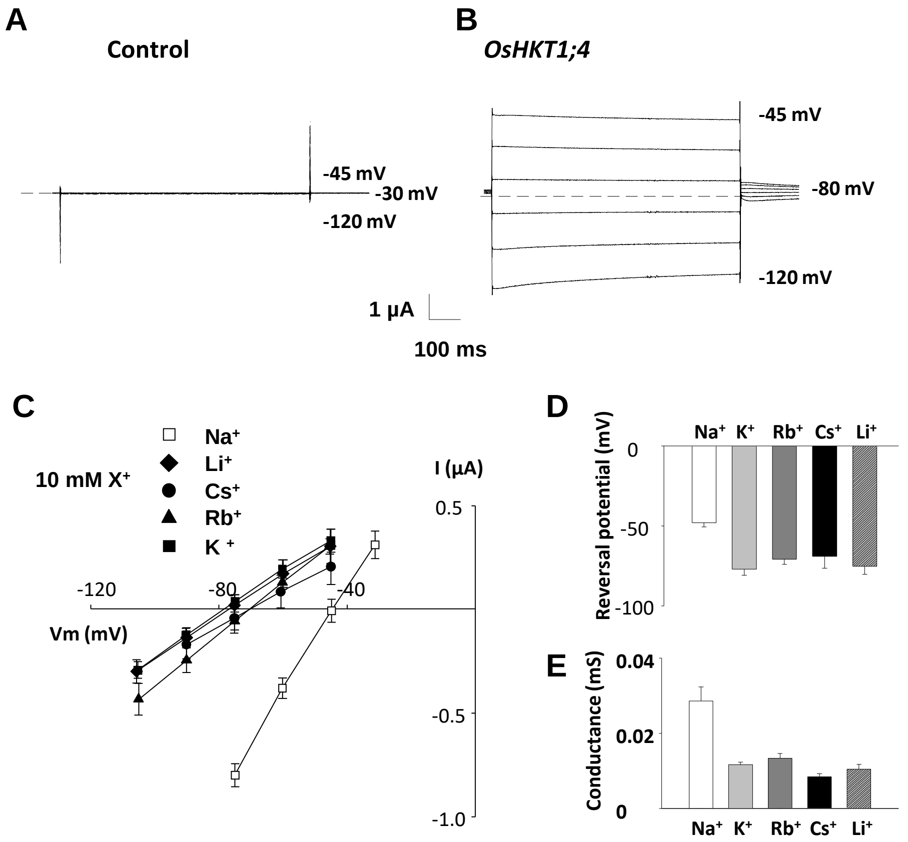

FIGURE S2 | OsHKT1;4 relative permeability and conductance between cations. (A, B) Representative current traces recorded in a control oocyte injected with 50 nl of water (A), and in an oocyte injected with 50 nl of *OsHKT1;4* cRNA (B) in a bath solution containing 3 mM Na^+ (as glutamate salt). The applied membrane voltage was stepped from -45 to -120 mV with _15 mV increments, from a holding potential set at -30 mV (A) or -80 mV (B). (C to E) OsHKT1;4 selectivity between monovalent cations. (C) OsHKT1;4 current-voltage (I-V) relationships in the presence of 10 mM of different cations (Na^+, K^+, Rb^+, Cs^+, Li^+), successively present (as Cl^- salts) in the bath solution. (D, E) Reversal potential of OsHKT1;4 currents (D) and OsHKT1;4 inward conductance (E) in the presence of the different cations. Analyzed oocytes are the same as in (C). Data in (C) to (E) are means ± SE (n = 3).
