# Supplementary material for: Constitutive Contribution by the Rice OsHKT1;4 Na+ Transporter to Xylem Sap Desalinization and Low Na+ Accumulation in Young Leaves Under Low as High External Na+ Conditions
Source: Front Plant Sci. 2020 Jul 30;11:1130. doi: 10.3389/fpls.2020.01130 (PMC7406799; doi:10.3389/fpls.2020.01130)
Supplement: Supplementary file 3 [file DataSheet_3.pdf]

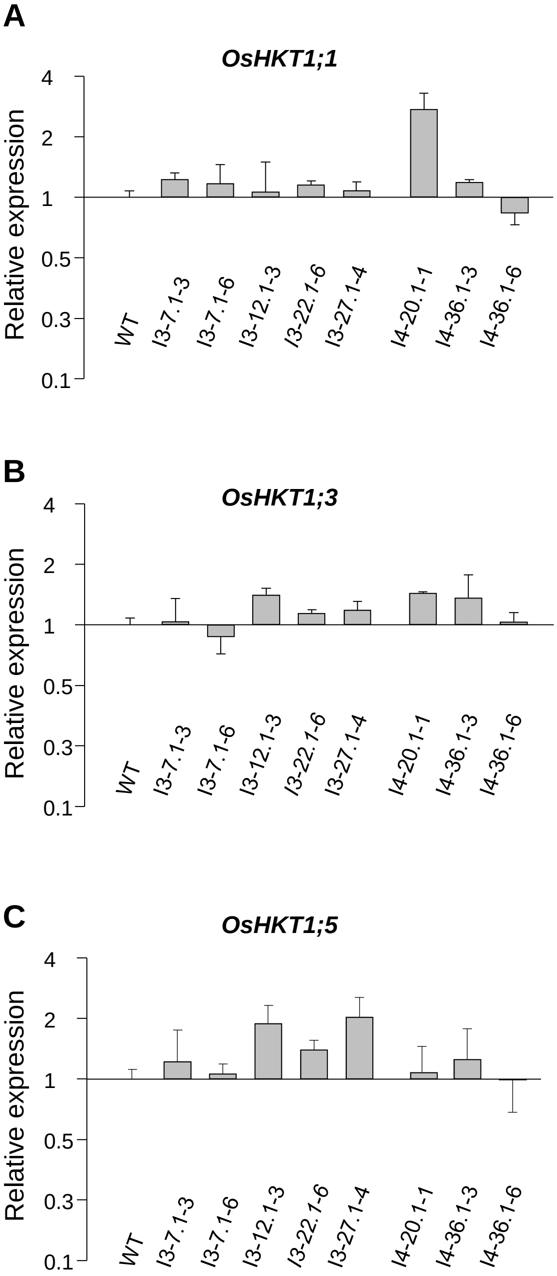

FIGURE S3 | Expression levels of *OsHKT1;1*, *OsHKT1;3* and *OsHKT1;5* in *oshkt1;4* amiRNA rice lines I3-# and I4-# from the T2 generation as compared to the corresponding expression levels in control wild type plants. Relative expression of *OsHKT1;1* **(A)**, *OsHKT1;3* **(B)** and *OsHKT1;5* **(C)**. Plants were the same as those analyzed for *OsHKT1;4* expression in Figure 5C. Control plants (WT) were issued from the same transformation as I3-# and I4-# lines, but did not express *PI3* or *PI4* microRNA precursors. The expression level of *elongation factor 1-beta* (*EF-1-beta*) was used as a constitutive calibration control in qRT-PCR experiments. Results from five plants issued from a same T1 plant were averaged (Means ± SE).
