# Supplementary material for: Constitutive Contribution by the Rice OsHKT1;4 Na+ Transporter to Xylem Sap Desalinization and Low Na+ Accumulation in Young Leaves Under Low as High External Na+ Conditions
Source: Front Plant Sci. 2020 Jul 30;11:1130. doi: 10.3389/fpls.2020.01130 (PMC7406799; doi:10.3389/fpls.2020.01130)
Supplement: Supplementary file 4 [file DataSheet_4.pdf]

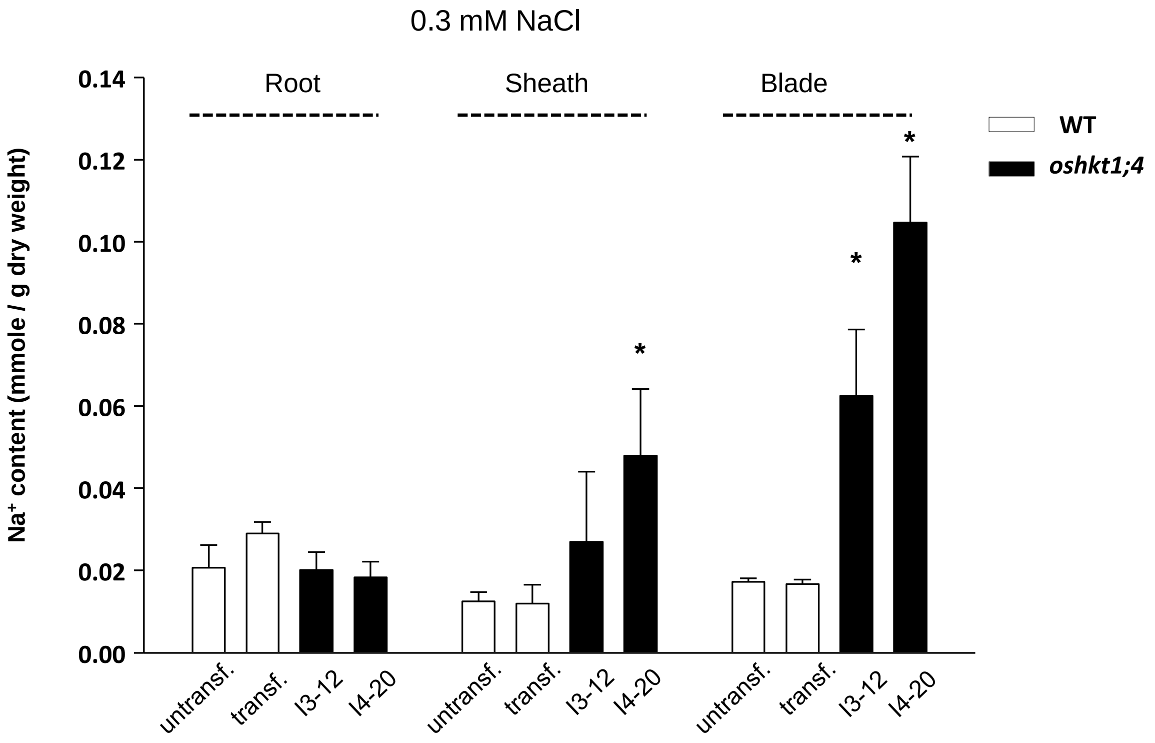

FIGURE S4 | Comparison of *oshkt1;4* amiRNA mutant lines and wild type controls with respect to sodium contents in roots and third leaf sheath and blade. *oshkt1;4* mutant plants displaying reduced expression of *OsHKT1;4* (Figure 5C) were issued from T2 generation of amiRNA I3 or amiRNA I4 lines (Figure 5A, B). Wild type plants (WT, cv Nipponbare) were either untransformed plants (untransf.) or plants transformed (transf.) with an empty pCambia5300 vector, the transformation having been carried out in parallel with that of the *oshkt1;4* amiRNA mutant lines (same calluses). Plants were hydroponically grown on Yoshida medium containing 0.3 mM Na^+ as described in Figure S1A. Na^+ contents were determined in the roots, the sheath of the third leaf and the blade of the third leaf. Means ± SE (n = 4 to 7). One star above a bar of mutant plants indicates that the difference with each type of wild type plants (untransf. and transf.) is statistically significant according to a Student t-test ($P \leq 0.05$). The data from the two types of WT plants were not statistically different according to a Student t-test ($P > 0.05$). The two lines of amiRNA plants were not statistically different according to a Student t-test ($P > 0.05$) either.
