# Supplementary material for: Constitutive Contribution by the Rice OsHKT1;4 Na+ Transporter to Xylem Sap Desalinization and Low Na+ Accumulation in Young Leaves Under Low as High External Na+ Conditions
Source: Front Plant Sci. 2020 Jul 30;11:1130. doi: 10.3389/fpls.2020.01130 (PMC7406799; doi:10.3389/fpls.2020.01130)
Supplement: Supplementary file 5 [file DataSheet_5.pdf]

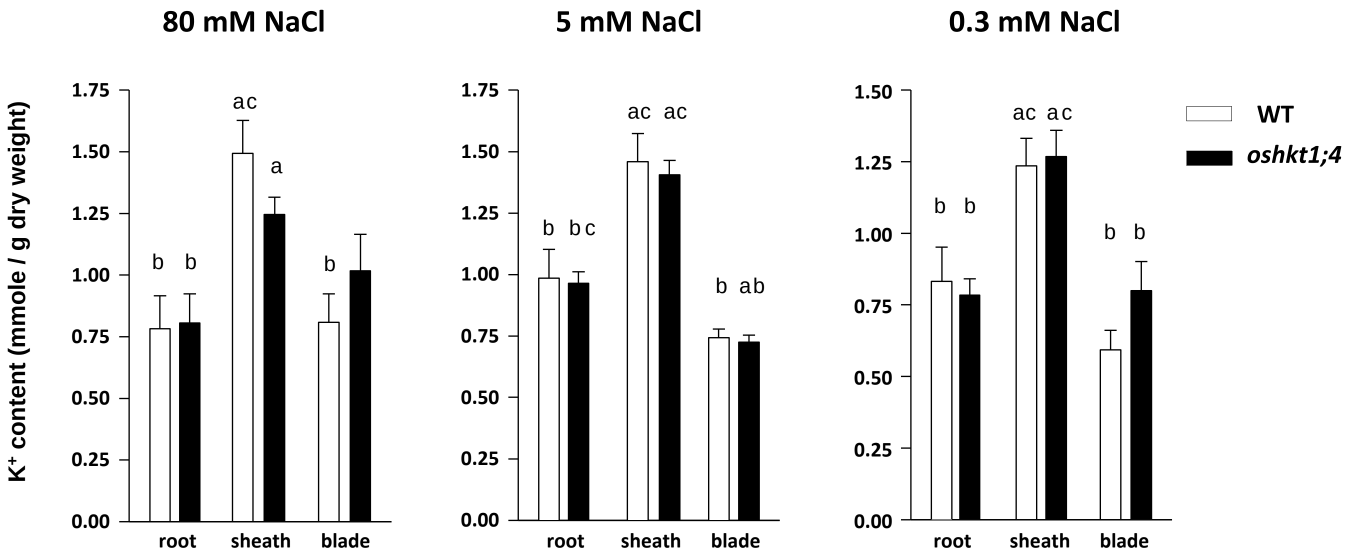

FIGURE S5 | Potassium contents in roots and leaf tissues of rice wild type and of *oshkt1;4* knockdown mutant plants treated with different levels of salt. Plants (same plants as those analyzed in Figure 6A) were grown and treated as described in Figure S1A. *oshkt1;4* mutant plants displaying reduced expression of *OsHKT1;4* (Figure 5C) were issued from T2 generation of amiRNA I3-# and I4-# lines. K^+ contents were determined in the roots, the sheath of the third leaf and the blade of the third leaf of plants treated with 80 mM NaCl (left panel), 5 mM NaCl (middle panel), or 0.3 mM NaCl (right panel). Means ± SE. Plants treated with 80 mM NaCl: n = 8 for the WT plants and 21 for the *oshkt1;4* mutant plants. Treatment with 5 mM NaCl: n = 7 and 13 for the WT and the *oshkt1;4* plants. Treatment with 0.3 mM NaCl: n = 11 and 21 for the WT and the *oshkt1;4* plants. No statistically significant difference (Student t-test, P>0.05) is found between the wild type (WT) and mutant plants. Within a same genotype, a letter above a bar (a, b or c) indicates significant difference (Student t-test, P ≤0.05) with another tissue (respectively root, leaf sheath or leaf blade).
