# Supplementary material for: Constitutive Contribution by the Rice OsHKT1;4 Na+ Transporter to Xylem Sap Desalinization and Low Na+ Accumulation in Young Leaves Under Low as High External Na+ Conditions
Source: Front Plant Sci. 2020 Jul 30;11:1130. doi: 10.3389/fpls.2020.01130 (PMC7406799; doi:10.3389/fpls.2020.01130)
Supplement: Supplementary file 6 [file Table_1.pdf]

Table S1. Selected amiRNA

	Selected amiRNA	Mismatch position on amiRNA	Hybridization energy (sequence similarity)	Target position on transcript (5' -> 3‘)
OsHKT1;4 amiRNA I3	TTAGTGACTAGGATAAGCGAT	1 and 19	-38.97 kcal/mol (93.52%)	1981-2001
OsHKT1;4 amiRNA I4	TTCTAACTAAGTTTCCAGGGT	1 and 20	-36.72 kcal/mol (89.10%)	1485-1505
