# Supplementary material for: Constitutive Contribution by the Rice OsHKT1;4 Na+ Transporter to Xylem Sap Desalinization and Low Na+ Accumulation in Young Leaves Under Low as High External Na+ Conditions
Source: Front Plant Sci. 2020 Jul 30;11:1130. doi: 10.3389/fpls.2020.01130 (PMC7406799; doi:10.3389/fpls.2020.01130)
Supplement: Supplementary file 7 [file Table_2.pdf]

Table S2. Primers for directed mutagenesis of pNW55 vector

Clone	Primer	Sequence	Alias	Type
all	Universal	GGGGacaagtttgtacaaaaaagcaggctCTGCAAGGCGATTAAGTTGGGTAAC	attB1G-4368	Forward
	Universal	GGGGaccactttgtacaagaaagctgggtGCGGATAACAATTTCACACAGGAAACAG	attB2G-4369	Reverse
OsHKT1;4 amiRNA I3	primer I	agTTAGTGACTAGGATAAGCGATcaggagattcagtttga	H7-A miR1	miR-s
	primer II	tgATCGCTTATCCTAGTCACTAActgctgctgctacagcc	H7-A miR2	miR-a
	primer III	ctATCGCATATGCTAGTCACTAAttcctgctgctaggctg	H7-A miR3	miR*-s
	primer IV	aaTTAGTGACTAGCATATGCGATagagaggcaaaagtgaa	H7-A miR4	miR*-a
OsHKT1;4 amiRNA I4	primer I	agTTCTAACTAAGTTTCCAGGGTcaggagattcagtttga	H7-B miR1	miR-s
	primer II	tgACCCTGGAAACTTAGTTAGAActgctgctgctacagcc	H7-B miR2	miR-a
	primer III	ctACCCTCGAATCTTAGTTAGAAttcctgctgctaggctg	H7-B miR3	miR*-s
	primer IV	aaTTCTAACTAAGATTCGAGGGTagagaggcaaaagtgaa	H7-B miR4	miR*-a

Lower case sequence in universal primers: *attB1* and *attB2* sites used for gateway cloning. Upper case sequence in the primers for cloning the amiRNA precursors: *amiR* and *amiR** sequences (*amiR* targeting *OsHKT1;4)* for replacement of the miRNA and miRNA* sequences of the naturally transcribed *osa-MIR528* already present in pNW55 vector.
