# Supplementary material for: Constitutive Contribution by the Rice OsHKT1;4 Na+ Transporter to Xylem Sap Desalinization and Low Na+ Accumulation in Young Leaves Under Low as High External Na+ Conditions
Source: Front Plant Sci. 2020 Jul 30;11:1130. doi: 10.3389/fpls.2020.01130 (PMC7406799; doi:10.3389/fpls.2020.01130)
Supplement: Supplementary file 8 [file Table_3.pdf]

Table S3. Primers used for real-time PCR expression analyses.

Gene	Primer name	Primer sequence and position / ATG	Amplicon length (bp)
OsHKT1;1	HIS_HKT4-F	$^{1421}5^{/}$-GGAAGTCAATTTCTTCTGATCCA-$3^{/}$	239*
	HIS_HKT4-R	$^{1660}5^{/}$-CAAGGAGTTCATCCTGAAATGA-$3^{/}$	
	QRT138H4F	$^{1492}5^{/}$-GGCAATGTAGGCTACTCACT-$3^{/}$	138
	QRT138H4R	$^{1631}5^{/}$-TATCCTGGTGATGTTCCTGG-$3^{/}$	
OsHKT1;3	H6B418F	$^{1324}5^{/}$-ATCACAGAACGGGACTCGAT-$3^{/}$	268*
	H6B418R	$^{1592}5^{/}$-TGGAAGAGCCTGGAAGCTTA-$3^{/}$	
	5???KT6a	$^{1340}5^{/}$-CGATGGCTACAGATCCACTT-$3^{/}$	127
	3???KT6a	$^{1467}5^{/}$-CTGAACCATGATGCACGCTG-$3^{/}$	
OsHKT1;4	H7B529F	$^{1396}5^{/}$-TAGGGAGATGGAGCGACTCT-$3^{/}$	424*
	H7B529R	$^{1820}5^{/}$-GTAAAAGGCCGGATGTCATT-$3^{/}$	
	QRT89H7F	$^{1448}5^{/}$-TGGGAGGCTCAAGAAGTTCA-$3^{/}$	89
	QRT89H7R	$^{1537}5^{/}$-ACCAGCTGAACATTGCAGGT-$3^{/}$	
OsHKT1;5	H8B388F	$^{1269}5^{/}$-CCAAGACAAACACCAGCAAA-$3^{/}$	300*
	H8B388R	$^{1568}5^{/}$-GTTGGCTTCTCAGGGAAGTG-$3^{/}$	
	QRT114H8F	$^{1363}5^{/}$-GCCTAGCCATCTTCATCGTC-$3^{/}$	114
	QRT114H8R	$^{1477}5^{/}$-GGTTATCAGTGCGTATGGCA-$3^{/}$	
SMT3	SMT3B303F	$^{14}5^{/}$-GGGAGGAGGACAAGAAGC-$3^{/}$	273*
	SMT3B303R	$^{286}5^{/}$-ATGCTCCACCAGACTGGAG-$3^{/}$	
	QRT94SMT3-F	$^{66}5^{/}$-CCTCAAGGTCAAGGGACAGG-$3^{/}$	94
	QRT94SMT3-R	$^{160}5^{/}$-TGAACGCCTATTGTGACCGT-$3^{/}$	
TIP 41	Tip41B389F	$^{187}5^{/}$-TGGTTTTTGGGGAGAGTTTC-$3^{/}$	389*
	Tip41B389R	$^{576}5^{/}$-TAGCCGATAATGGCATTTCA-$3^{/}$	
	QRT101Tip41-F	$^{392}5^{/}$-TGGGAGTGATGCTTTGGTTC-$3^{/}$	101
	QRT101Tip41-R	$^{493}5^{/}$-TGAGGATCGGATTGACCTTG-$3^{/}$	
eEF-1-β2	eEFB2Pcs	$^{332}5^{/}$-TGGTGAGGAGACTGAAGAGG-$3^{/}$	418*
	eEFB2Pcas	$^{750}5^{/}$-CTTCCGGATTTTTCTTTTTATC-$3^{/}$	
	QRT151eE	$^{476}5^{/}$-GGAAGAAGCTGTGAGGAATG-$3^{/}$	176
	QRT151eEF&# 946;2-R	$^{652}5^{/}$-CGAACCAGCGAATGAGTACA-$3^{/}$	

*Larger amplicons, which were used as a template for the amplification of calibration standards.
