# Supplementary material for: Constitutive Contribution by the Rice OsHKT1;4 Na+ Transporter to Xylem Sap Desalinization and Low Na+ Accumulation in Young Leaves Under Low as High External Na+ Conditions
Source: Front Plant Sci. 2020 Jul 30;11:1130. doi: 10.3389/fpls.2020.01130 (PMC7406799; doi:10.3389/fpls.2020.01130)
Supplement: Supplementary file 9 [file Table_4.pdf]

Table S4. Primers used for *OsHKT1;4 promoter::GUS* construct

Primer name	Primers sequence (5’-3’)	Amplified sequence length	Cloned promoter length
GOsHKT7F1	**AGCTAGCAGCAGAGGAAAACGATG**	**2489 bp**	**2175 bp**
GOsHKT7RM2	**GCGAGCGCGCGCCGCGACGTGGCCATGGG**		
